# Treatment with the senolytics dasatinib/quercetin reduces SARS‐CoV‐2‐related mortality in mice

**DOI:** 10.1111/acel.13771

**Published:** 2023-01-26

**Authors:** Andrés Pastor‐Fernández, Antonio R. Bertos, Arantzazu Sierra‐Ramírez, Javier del Moral‐Salmoral, Javier Merino, Ana I. de Ávila, Cristina Olagüe, Ricardo Villares, Gloria González‐Aseguinolaza, María Ángeles Rodríguez, Manuel Fresno, Nuria Gironés, Matilde Bustos, Cristian Smerdou, Pablo Jose Fernandez‐Marcos, Cayetano von Kobbe

**Affiliations:** ^1^ Metabolic Syndrome Group‐BIOPROMET Madrid Institute for Advanced Studies‐IMDEA Food, CEI UAM+CSIC Madrid Spain; ^2^ Department of Internal Medicine and Surgical Animal, Faculty of Veterinary/VISAVET Centre Complutense University of Madrid Madrid Spain; ^3^ Departamento de Biología Molecular Universidad Autónoma de Madrid (UAM) Madrid Spain; ^4^ Centro de Biología Molecular Severo Ochoa (CSIC‐UAM) Consejo Superior de Investigaciones Científicas (CSIC) Madrid Spain; ^5^ Centro de Investigación Biomédica en Red de Enfermedades Hepáticas y Digestivas (CIBERehd) del Instituto de Salud Carlos III Madrid Spain; ^6^ Division of Gene Therapy and Regulation of Gene Expression CIMA Universidad de Navarra Pamplona Spain; ^7^ Centro Nacional de Biotecnología (CNB‐CSIC) Consejo Superior de Investigaciones Científicas (CSIC) Madrid Spain; ^8^ Institute of Biomedicine of Seville (IBiS), Spanish National Research Council (CSIC) University of Seville, Virgen del Rocio University Hospital Seville Spain

**Keywords:** cellular senescence, COVID‐19, SARS‐CoV‐2, senolytics, survival

## Abstract

The enormous societal impact of the ongoing COVID‐19 pandemic has been particularly harsh for some social groups, such as the elderly. Recently, it has been suggested that senescent cells could play a central role in pathogenesis by exacerbating the pro‐inflammatory immune response against SARS‐CoV‐2. Therefore, the selective clearance of senescent cells by senolytic drugs may be useful as a therapy to ameliorate the symptoms of COVID‐19 in some cases. Using the established COVID‐19 murine model K18‐hACE2, we demonstrated that a combination of the senolytics dasatinib and quercetin (D/Q) significantly reduced SARS‐CoV‐2‐related mortality, delayed its onset, and reduced the number of other clinical symptoms. The increase in senescent markers that we detected in the lungs in response to SARS‐CoV‐2 may be related to the post‐COVID‐19 sequelae described to date. These results place senescent cells as central targets for the treatment of COVID‐19, and make D/Q a new and promising therapeutic tool.

## Graphical abstract

New treatments are necessary to reduce the social impact of COVID‐19. Using the murine model K18‐hACE2, we demonstrated that senolytics (D/Q) reduced SARS‐CoV‐2‐related mortality, delayed its onset, and reduced the number of other clinical symptoms.

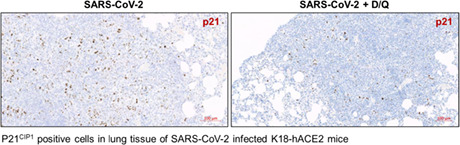

## 1 INTRODUCTION

The COVID‐19 pandemic, caused by SARS‐CoV‐2 (Zhu et al., 2019), has affected more than 620 million people worldwide, resulting in more than 6.5 million deaths to date (WHO, n.d.). The most severely affected include the elderly and people with certain pathologies such as hypertension and cardiovascular diseases, diabetes, chronic lung diseases, cancer and immunosuppression, collectively comprising the highest COVID‐19 risk groups (Huang et al., 2020a; Palaiodimos et al., 2020; Ruan et al., 2020). New treatments are necessary to reduce the social impact of this disease, both in terms of mortality and morbidity.

SARS‐CoV‐2 promotes a systemic inflammatory sequence of events across the body, mainly driven by myeloid‐lineage cells in the peripheral blood, lungs and airways. Infected cells promote the production of pro‐inflammatory mediators that lead to a systemic activation of monocytes and macrophages (Daamen et al., 2021).

It has been proposed that senescent cells may play a central role in the development of severe COVID‐19 disease (Blagosklonny, 2020; Camell et al., 2021; Kirkland & Tchkonia, 2020; Malavolta et al., 2020; Mohiuddin & Kasahara, 2021; Nehme et al., 2020). In fact, the chronic accumulation of senescent cells causes several diseases, most of them also associated with the same COVID‐19 risk groups.

Here, we used an established model for the in vivo study of COVID‐19 comprised of the causal pathogen, SARS‐CoV‐2 (Zhu et al., 2019), and K18‐hACE2 transgenic mice (Golden et al., 2020; Oladunni et al., 2020; Rathnasinghe et al., 2020; Winkler et al., 2020; Yinda et al., 2021; Zheng et al., 2020). This transgenic murine model was originally developed for SARS‐CoV infection studies (McCray et al., 2007). These mice express under the cytokeratin promotor, the main SARS‐CoV‐2 receptor, the human angiotensin‐converting enzyme 2 (hACE2), in the airway ephitelial cells (among others organs) (McCray et al., 2007). Upon SARS‐CoV‐2 infection, these mice recapitulate most of the human COVID‐19 symptoms (Golden et al., 2020; Oladunni et al., 2020; Rathnasinghe et al., 2020; Winkler et al., 2020; Yinda et al., 2021; Zheng et al., 2020).

We demonstrated that the senolytic combination of D/Q significantly reduced SARS‐CoV‐2‐related mortality, in addition to reducing other clinical symptoms. Upon D/Q treatment, we also detected a cytokine pattern that has been associated with mild disease in other reports. Importantly, we observed a time‐dependent accumulation of four senescence markers in lung tissue in response to SARS‐CoV‐2, which was reduced by D/Q treatment. These results place senescent cells as central targets for treatment of COVID‐19, and suggest the promise of novel therapies based on anti‐aging compounds.

## 2 RESULTS

### 2.1 Evaluation of senolytic treatment for COVID‐19

**FIGURE 1:**
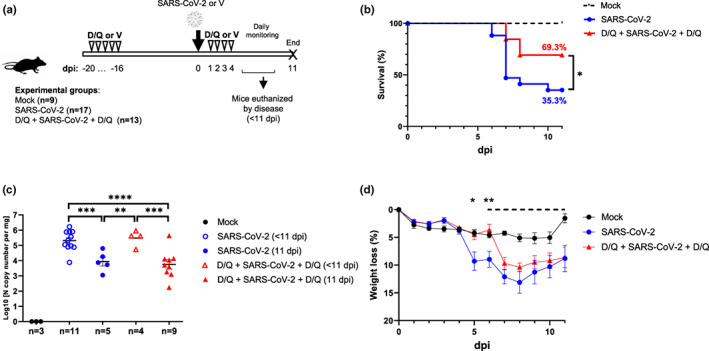
Experimental scheme and impact of SARS‐CoV‐2 in K18‐hACE2 mice. (a) Experimental design for the animal study. Ten‐month‐old K18‐hACE2 mice (males) were divided into three groups, treated with either D/Q, SARS‐CoV‐2 or vehicle (V), and monitored as indicated in Methods. Dpi: Days post‐infection. Negative numbers indicate days pre‐infection (preventive therapy). (b) Percentage survival of mice. *p* Value was determined by Gehan–Breslow–Wilcoxon test (*p = 0.0398). (c) Viral RNA levels in lungs analyzed by RT–qPCR. *p* Values were determined by one‐way ANOVA, Fisher's LSD test (**p = 0.0015; ***p ≤ 0.0005; ****p < 0.0001). (d) Weight change (±SEM) relative to day 0 was monitored (*N* of each group as indicated in [a]). The dashed line indicates the start of SARS‐CoV‐2‐induced deaths, and thus changing the *N* for each group. *p* Values were determined by two‐way ANOVA (uncorrected Fisher's LSD; mixed‐effects analysis) test (*p = 0.0412; **p = 0.0054). Data are combined from two independent experiments.

Our first objective was to determine the optimal SARS‐CoV‐2 dose in vivo, which was 10^4^ PFU/mouse (Figure S1a–d). This dose has also been previously described (Zheng et al., 2020), thus validating our disease severity model. To demonstrate the central role of senescent cells in the severity of COVID‐19, we first evaluated a preventive/therapeutic treatment (hereafter, D/Q + SARS‐CoV‐2 + D/Q) using the senolytic drugs D/Q, in a SARS‐CoV‐2 challenge experiment (Figure 1a). Mice were divided into three groups: Mock; SARS‐CoV‐2 and D/Q + SARS‐CoV‐2 + D/Q. Once infected, mice were monitored daily until day 11 dpi, the scheduled endpoint of the experiment.

The presence of viral RNA was analyzed at 4 dpi in nasal swabs as infection control. As expected, all mice in the infected group (and none in the mock‐infected) were PCR positive (Figure S2).

Importantly, we observed significant differences in mortality kinetics between the groups (Figure 1b). In the group only infected with SARS‐CoV‐2, 11 mice were euthanized by end‐point criteria before the scheduled end of the experiment (<11 dpi), corresponding to a mortality rate of 64.7% (11/17). In contrast, in the group treated with D/Q, only four mice had to be euthanized early (at 7 and 8 dpi), with a mortality rate of only 30.7% (4/13). According to these results, SARS‐CoV‐2 infection resulted in two clear sub‐groups: those that were euthanized and/or died before the end‐point (mostly at 7–8 dpi), and those that survived until the scheduled end of the experiment (11 dpi). This distinction is important since SARS‐CoV‐2 replication follows a time‐dependent kinetic, and we will present our findings according to these two groups from now on.

Therefore, a preventive/therapeutic D/Q treatment significantly reduced SARS‐CoV‐2‐induced mortality in K18‐hACE2 mice, illustrating a protective effect for the combination of senolytics D/Q in an experimental COVID‐19 setting.

### 2.2 Analysis of viral RNA levels in lungs

Lungs are one of the predominant target organs of SARS‐CoV‐2. When we analyzed the presence of viral RNA in the lungs, we observed the highest levels in those mice euthanized early (<11 dpi; Figure 1c). Surviving mice from both the infected and infected + D/Q treatment groups had significantly lower levels of viral RNA. This effect was more evident in the D/Q‐treated group, since more survived (9/13; 69.3%) compared to the infected, untreated group (6/17; 35.3%). The differences in viral RNA levels were not due to higher initial viral infection, as swabs obtained at 4 dpi showed no significant viral RNA differences, even when considering which mice were sacrificed early (<11 dpi) (Figure S2).

Thus, high SARS‐CoV‐2 RNA levels in the lungs were significantly associated with increased mortality, and D/Q treatment significantly increased the number of mice with reduced viral RNA burden.

The expression of SARS‐CoV‐2 N protein was detected over time in lung tissue from infected mice using immunohistochemistry (IHC), with a pattern of expression consistent with the RNA levels shown above. We observed staining in bronchioles, as well as patchy areas with strong staining in alveoli (perhaps associated with dead cells) (Figure S3a). The staining was more intense, with significantly greater numbers of foci/regions expressing N protein, in the lungs of early‐euthanized mice (<11 dpi), compared to the survivors (Figure S3b), in agreement with previous data showing strong SARS‐CoV‐2 N signal in the first week of infection (Yinda et al., 2021; Zheng et al., 2020).

### 2.3 Evaluation of disease‐associated symptoms

Over the course of the study, onset of the characteristic symptoms of SARS‐CoV‐2 infection were analyzed, including weight loss (Figure 1d). The mean change in weight per experimental group showed that until 6 dpi there was hardly any weight loss in either the mock group or the group treated with D/Q. In contrast, the SARS‐CoV‐2‐infected, untreated group exhibited an evident and significant loss of weight starting at 4–5 dpi. From 7 dpi, weight loss similarly affected both groups infected with SARS‐CoV‐2, regardless of D/Q treatment. When we analyzed the individual mouse data (Figure S4), it was clear that through 4–6 dpi, all the mice treated with D/Q showed a weight change kinetic that was practically identical to the mock‐infected group, and clearly different from SARS‐CoV‐2‐infected, the untreated group. Subsequently (from 7 dpi), the weight loss in both infected groups was very similar.

Therefore, treatment with the senolytics D/Q delayed SARS‐CoV‐2‐associated weight loss by 2 days.

**FIGURE 2:**
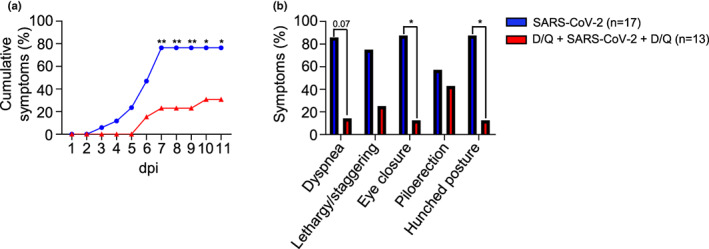
Senolytics reduce the onset of SARS‐CoV‐2‐related symptoms. The analyzed symptoms (excluding weight loss) consisted of dyspnea, lethargy and staggering, eye closure, piloerection, and hunched posture. Mock‐infected mice did not exhibit any symptoms throughout the experiment. (a) The number of cumulative symptoms, exhibiting at least one of the previously described, are represented for each group. (b) The individual symptoms are considered as positive when a mouse showed it at any day during the period 1–11 dpi. *p* Values were determined using the chi‐square test of independence. *p ≤ 0.05; **p ≤ 0.005. Data are combined from two independent experiments.

Next, we assessed other clinical symptoms that have previously been described in response to SARS‐CoV‐2, such as dyspnea, lethargy/staggering, eye closure, piloerection and hunched posture. Mice belonging to the SARS‐CoV‐2 group accumulated an elevated number of symptoms between 7 and 11 dpi (Figure 2a). Treatment with D/Q delayed the onset of these symptoms for 3 days resulting in a 45% decrease in cumulative symptoms at the end of the assay (Figure 2a). The specific symptomatology showed a significant reduction of eye closure and hunched posture, and a tendency of lower dyspnea when mice were treated with D/Q. None of these symptoms were detected in the mock group (Figure 2b). Most of the analyzed symptoms were associated with mortality, as they were detected in the mice that were euthanized early (<11 dpi).

All together, these results show that preventive/therapeutic D/Q treatment delayed the onset of symptoms associated with SARS‐CoV‐2 infection, in addition to significantly reducing the number of eye closure and hunched posture. Importantly, the tendency of lower dyspnea is related with a better lung function.

### 2.4 Serum levels of cytokines and chemokines

SARS‐CoV‐2 produces an inflammatory status in several tissues and is associated to a monocyte and macrophage activation (Daamen et al., 2021). In addition, in senescent cultured cells, the SARS‐CoV‐2 spike protein has been previously shown to amplify the senescence‐associated secretory phenotype (SASP), characterized by increased pro‐inflammatory factors such as *Ifnγ, Il1α, Il1β, Il6, Il17, Tnfα, Cxcl1, Cxcl2, Cxcl10, Mcp1, Mip1, Pai1, Pai2, Il2*, and *Il7* (Camell et al., 2021).

**FIGURE 3:**
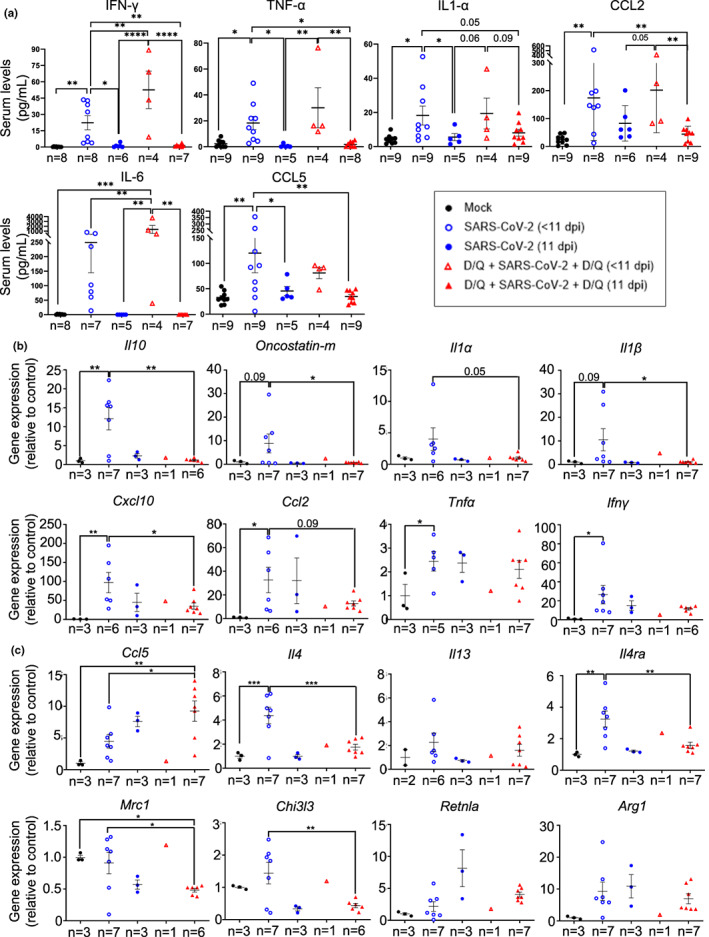
Cytokine and chemokine levels in serum and lungs. (a) Multiplex platform was used to measure the serum levels of the indicated proteins in each experimental group (*n* as indicated in the graphs). Data are combined from two independent experiments. (b, c) mRNA expression of cytokines and chemokines associated with severe SARS‐CoV‐2 disease (b) or associated with Th2 cytokines and monocyte/macrophage function (c) in lungs of the indicated mouse groups. *p* Values were determined by one‐way ANOVA, Fisher's LSD test. *p ≤ 0.05; **p ≤ 0.005; ***p ≤ 0.0005; ****p ≤ 0.0001. Representative data from one out of two independent experiments.

Therefore, we aimed to study the ability of senolytic drugs to reduce the SASP phenotype and its implications in cytokine‐mediated immune recruitment. First, to investigate the systemic levels of cytokines and chemokines, we used a pre‐configured multiplex panel. We detected significantly higher levels of the pro‐inflammatory cytokines IFN‐γ, TNF‐α, IL‐1α, CCL‐2 (MCP‐1), CCL‐5 (Rantes), and IL‐6 in all the infected mice that had to be euthanized before 11 dpi (Figure 3a). These data agree with previous reports, associating higher levels of these cytokines with mortality (Huang et al., 2020b; Yang et al., 2020; Ye et al., 2020), and also described in the serum of this murine model upon SARS‐CoV‐2 infection (Golden et al., 2020; Yinda et al., 2021). Importantly, survival at 11 dpi was associated with reduced levels of these pro‐inflammatory factors (Figure 3a).

The increased levels of CCL2 (also called MCP‐1), IL‐1α, or CCL‐5 (Rantes) in the euthanized mice before 11 dpi suggest a systemic inflammatory response, again associated with mortality (Figure 3a). No signal was detected when analyzing IL‐10 and GM–CSF (data not shown).

Thus, D/Q treatment significantly increased the number of mice with reduced levels of pro‐inflammatory cytokines associated with the COVID‐19‐related mortality.

### 2.5 mRNA expression of cytokines and chemokines in lungs

In addition, we measured mRNA expression of cytokines and chemokines specifically in lungs. SARS‐CoV‐2‐infected mice showed increased levels of *Il10* (Islam et al., 2021), *Oncostatin‐m* (Oladunni et al., 2020), *Il1α* (Ling et al., 2021), *Il1β* (Oladunni et al., 2020), *Cxcl10* (Oladunni et al., 2020), *Ccl2* (Oladunni et al., 2020), *Tnfα* (Oladunni et al., 2020), and *Ifnγ* (Oladunni et al., 2020) in their lungs, which are related to disease progression (Figure 3b). These data indicate that the infection is under the second period of cytokine production (Chen et al., 2010), which fits with the time of sampling (between 7 and 11 dpi). Mice surviving until 11 dpi, both treated with vehicle or with D/Q, showed a reduced expression of SARS‐CoV‐2‐associated markers *Il10*, *Oncostatin‐m*, *Il1α*, *Il1β*, *Cxcl10* and *Ccl2* (Figure 3b). On the contrary, *Tnfα* and *Ifnγ* mRNA expression was not decreased in the lungs (Figure 3b), although their levels did decrease in the serum (Figure 3a). We observed significant differences in the mRNA levels of most cytokines and chemokines, between the SARS‐CoV‐2 (<11 dpi) and D/Q + SARS‐CoV‐2 + D/Q (11 dpi) groups (Figure 3b), although the fact that they are samples from different post‐infection time‐points makes their comparison difficult. Likewise, no significant conclusions could be reached with the sub‐group D/Q + SARs‐CoV‐2 + D/Q (<11 dpi), because only one mouse died before the end‐point (i.e., *n* = 1).

These data indicate that our senolytic treatment increased the percentage of mice that had a reduction in inflammatory markers associated with disease progression, and are in line with the milder virus‐derived symptoms presented by this group and their enhanced survival rate (Figure 2).

Next, we focused on CCL5, a chemokine that attracts mainly monocytes (Schall et al., 1990), and participates in macrophage (Lee et al., 2017) and NK migration (Maghazachi et al., 1994). Elevated lung expression of CCL5 in SARS‐CoV‐2 patients has been associated with a milder COVID‐19 disease (Zhao et al., 2020). At 11 dpi, D/Q‐treated mice showed a tendency to increased *Ccl5* expression, compared with vehicle‐treated infected mice, although at this time point the difference was not significant (Figure 3c). We also analyzed additional markers regulating monocyte and macrophage function. IL4‐producing Th2 cells enhance anti‐inflammatory M2 macrophage differentiation, which in turn generate fibrosis and DAD (Boumaza et al., 2021; Vaz de Paula et al., 2020). The SARS‐CoV‐2‐infected, untreated mice who were euthanized early (<11 dpi) had increased lung expression of the Th2‐specific cytokine Il4 (Oladunni et al., 2020) (but not *Il13* (Oladunni et al., 2020), another Th2‐specific cytokine) and their receptor *Il4ra* (McCormick & Heller, 2015), and surviving animals (sacrificed at 11 dpi) belonging to both SARS‐CoV‐2‐infected groups showed decreased levels, regardless of D/Q treatment (Figure 3c). As the pattern of expression from *Il4* changed similarly as *Il4ra* (Figure 3c) we hypothesized that the survival‐related response (which was higher percentage‐wise in the D/Q‐treated group) reduced their IL4‐IL4Ra axis to diminish disease progression. We also observed a survival‐related reduction (again, more highly represented in the D/Q‐treated group) in additional type 2 markers regulated by IL4 or IL13, such as *Mrc1* (CD206) (Donlan et al., 2021), *Chi3l3* (Ym1) (Donlan et al., 2021) (Figure 3c) and *Il10* (Figure 3b), while other Il4 targets, such as *Retnla* (Fizz1) (Donlan et al., 2021) and *Arg1* (Donlan et al., 2021), remained unchanged (Figure 3c).

These data indicate that senolytic treatment promoted survival parameters related to a reduction of SARS‐CoV‐2 markers associated with Th2 and M2 macrophage differentiation, and suggest that the IL4‐IL4Ra axis is partly responsible for the beneficial effects of D/Q treatment.

### 2.6 Histopathological survey

**FIGURE 4:**
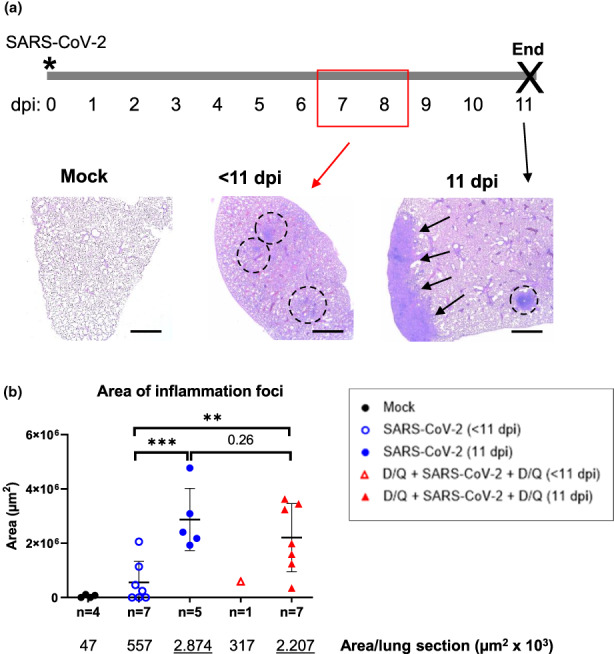
Lung injury. (a) Hematoxylin and eosin staining of representative lung sections from K18‐hACE2 mice. Left: Mouse lung section from the mock‐infected group. Small isolated lymphocyte accumulations were detected in 2 of 4 mice (not shown here; see Figure 5a). Middle: SARS‐CoV‐2‐infected mouse lung, euthanized before the scheduled end of the experiment (<11 dpi). Five of eight mice showed multifocal foci of inflammation (circles). Right: SARS‐CoV‐2‐infected mouse lung, sacrificed at the end of the experiment (11 dpi). The photo shows a 20% lung consolidation due to coalescence of numerous well‐defined foci (arrows). An average of 6–7 foci per lung were detected. Scale bars: 2000 μm. (b) Quantification of areas of dense inflammation foci (SARS‐CoV‐2‐treated mice) and lymphocyte accumulation (mock group), using ImageJ/FIJI software. Below is the numerical data of the area means per group of mice. *p* Values were determined by one‐way ANOVA, Fisher's LSD test. **p ≤ 0.005; ***p ≤ 0.0005. Data from one out of two independent experiments.

Histological study revealed different grades of pulmonary injury in the three experimental groups. (Figure 4a). In the case of the mock‐infected control group, the lungs of the four mice presented a normal histology.

The SARS‐CoV‐2‐infected, untreated animals that died at 7 dpi showed a shock lung characterized by an intense alveolar edema and hemorrhage with severe capillary septal congestion. In addition, there was moderate to severe bronchiolo‐interstitial pneumonia, with extensive and massive infiltration of lymphocytes at the level of the peribronchiolar interstitium and the alveolar septum. The distribution of the lesions was multifocal with a tendency to confluency. This process was delimited by a subacute pneumonic inflammatory process at the level of the alveolar lumen with the presence of lymphocytes, neutrophils, alveolar macrophages and numerous syncytia. Pericanalicular and subpleural foci with marked fibrosis were seen where there was proliferation of fibroblasts and deposits of connective tissue interspersed with lymphocytes and plasma cells. In these foci, there was an attempt to re‐epithelialize type II pneumocytes. Hyaline emboli were observed in the lumen of the blood vessels, which was a sign of disseminated intravascular coagulation (DIC).

Two infected animals that died at 8 dpi (one from the D/Q‐treated group, the other untreated) had a very similar picture to that described above. Nevertheless, it is important to note the histopathological findings of the infected, untreated mouse that died at 10 dpi, where, in addition to the shock lung, signs of vasculitis were seen. There were areas of suppurative pneumonia, in a patchy (multifocal) pattern with a tendency to confluency.

Infected mice who survived to the end of the experiment showed some differences. Those in the group not treated with D/Q, presented a moderate to severe lymphocytic, multifocal, chronic bronchiole‐interstitial pneumonia. This process consists of a chronic inflammatory infiltrate of round cells (mainly lymphocytes, plasma cells and some histiocytes) of moderate to intense degree with pericanalicular and perivascular distribution. There were areas of desquamative alveolitis with the presence of some neutrophils, macrophages and syncytial cells in a multifocal pattern. The surviving mice of the D/Q + SARS‐CoV‐2 + D/Q group displayed a more moderate interstitial pattern with marked involvement of the pleura. Three animals of this group showed severe pleural lesions, with some perivascular lymphocytic cuffs observed.

Thus, lung histopathology revealed different lesions and intensity according to the date of sacrifice (early vs. 11 dpi). Importantly, less severe lung injury was detected in the surviving D/Q‐treated mice (sacrificed on 11 dpi).

In fact, this tendency toward less lung damage upon treatment with D/Q was found when the areas of intense inflammation foci, produced by the coalescence of numerous individual foci, were quantified (Figure 4b). In this analysis, we observed 20% less inflammation foci area in the infected mice treated with senolytics compared to the untreated infected group. However, the difference was not significant, due to the marked inflammation indicated above, in three animals of the D/Q‐treated group.

### 2.7 Senescence marker levels in lungs upon SARS‐CoV‐2 infection, and effect of senolytics

**FIGURE 5:**
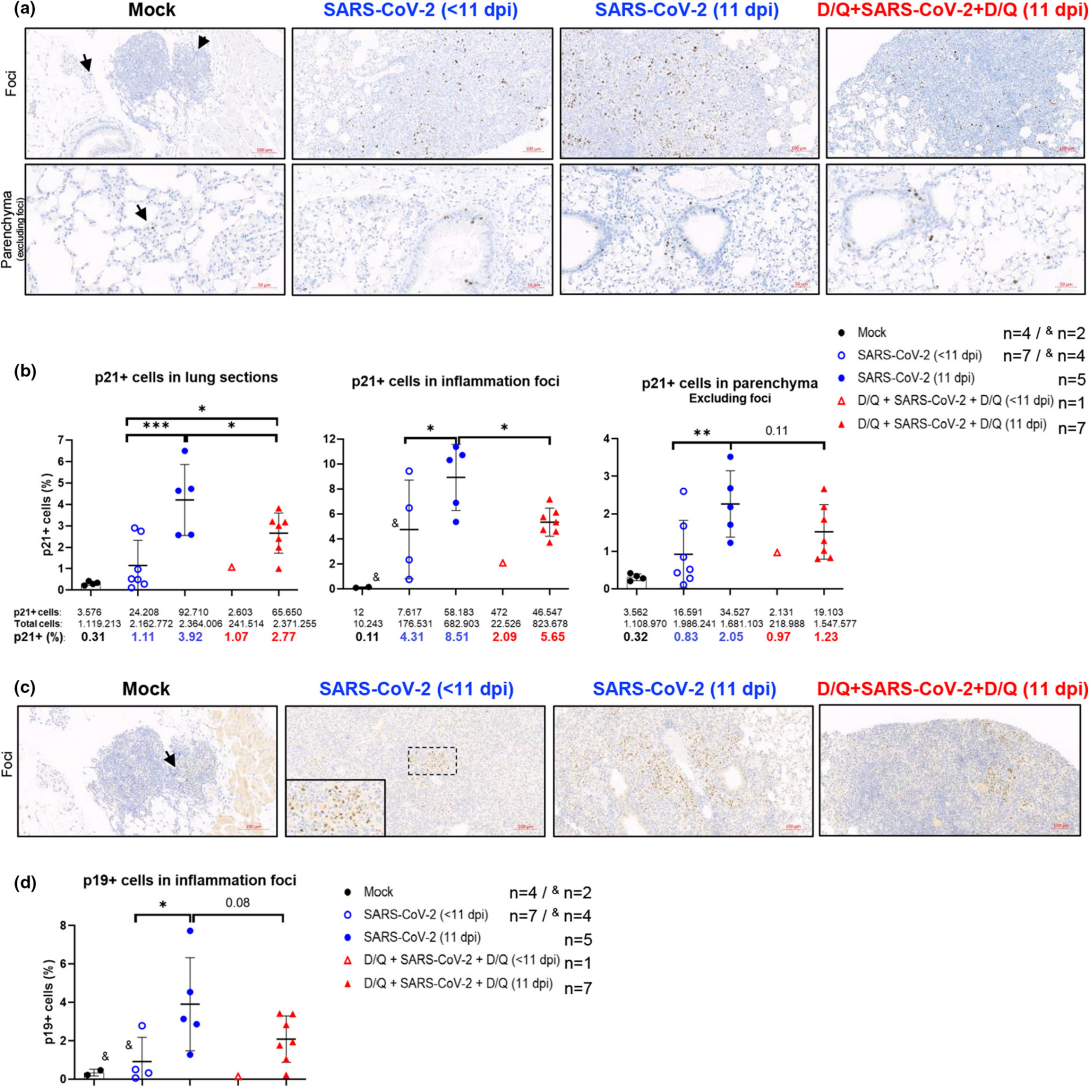
Immunohistochemistry (IHC) images of senescence markers in lung tissue from SARS‐CoV‐2 infected K18‐hACE2 mice. (a) Representative IHC of p21^CIP1^‐positive cells in the lungs of the indicated groups. Top row: Staining in isolated areas of lymphocyte accumulation (mock group), and in dense inflammation foci (SARS‐CoV‐2‐infected mice). Bottom row: Staining in the lung tissue (excluding inflammation foci), indicated as parenchyma. Arrows indicate p21^CIP1^‐positive cells in mock samples. Scale bars: 100 μm (top row) and 50 μm (bottom row). (b) Quantification of p21^CIP1^‐positive cells in whole lung sections (left graphic), only in inflammation foci (middle) and parenchyma (right graphic), using ImageJ/FIJI software. In the mock‐infected group, two of four animals displayed a few minor inflammation foci, as shown in the IHC. In the SARS‐CoV‐2 group, four of seven animals showed dense inflammation foci. Bottom numbers: Numerical data of the number of cells analyzed. *p* Values determined by one‐way ANOVA, Fisher's LSD test. *p ≤ 0.05; **p ≤ 0.005; ***p ≤ 0.0005. (c) Representative IHC of p19^ARF^‐positive cells in dense inflammation foci in lungs of the indicated mice groups. Arrows indicate p19^ARF^‐positive cell in mock sample. Scale bars: 100 μm (20 μm in the magnified square). (d) Quantification of p19^ARF^‐positive cells in dense inflammation foci of lung samples from individually analyzed mice, as described in b. *p* value determined by one‐way ANOVA, Fisher's LSD test. *p ≤ 0.05. Data from one out of two independent experiments.

Next, we analyzed by IHC the expression in the lung of three senescence markers, p21^CIP1^, p19^ARF^, and p16^INK4a^ (Figure 5a,c, Figure S7a), and subsequently the staining was quantified using an ad‐hoc designed macro (Figure 5b,d, Figure S7b,c). The data from whole lung sections showed that although expression of p21^CIP1^ was very low in the control mock‐infected mice (0.31% of p21^CIP1^‐positive cells), it increased in response to SARS‐CoV‐2 infection, with up to 1.1% in the mice euthanized early (<11 dpi), and even more, and significantly, up to 3.92% in the mice that survived until the end of the experiment (11 dpi) (Figure 5b). Importantly, D/Q treatment significantly reduced the percentage of p21^CIP1^‐positive cells to 2.77% in surviving mice, demonstrating the in vivo efficacy of senolytics treatment. In accordance, *p21* mRNA expression was also induced upon SARS‐CoV‐2 infection (at <11 dpi) (Figure S5), but D/Q treatment blunted this increase, although the fact of having only one sample (*n* = 1) in this experimental sub‐group, makes it difficult to draw robust conclusions. It is also important to note that at longer post‐infection times (11 dpi), there is an inverse correlation between *p21* mRNA and p21^CIP1^ protein levels. We cannot explain this discrepancy between the RT‐qPCR and IHC data, and whether SARS‐CoV‐2 could be implicated in p21^CIP1^ protein stability.

Next, we assessed whether the differences in expression in p21^CIP1^ were linked to a specific area of the lung. For this, we separately quantified dense inflammation foci (hereafter “foci”) and the rest of the lung (hereafter “parenchyma”) (Figure 5a). Importantly, in the foci we observed a robust increase in p21^CIP1^‐positive cells in response to SARS‐CoV‐2 infection. In this analysis we included a few isolated lymphocyte accumulations detected in two control mice (Figure 5a), which displayed only 0.11% of cells positive for p21^CIP1^ (Figure 5b). In stark contrast, the infected non‐survivors (euthanized <11 dpi) displayed 4.3% of cells positive for p21^CIP1^, and this number significantly increased to 8.5% in the mice who survived to the end of the study (11 dpi). Again, D/Q treatment significantly reduced the number of p21^CIP1^‐positive cells to 5.65% in the seven mice sacrificed at 11 dpi (Figure 5b).

The parenchyma data showed a similar pattern, with a time‐dependent increase in p21^CIP1^‐positive cells in the infected, untreated group (0.83% and 2.05% in the mice euthanized early [<11 dpi] or survivors, respectively). D/Q treatment reduced this number (2.05%) to 1.23%.

The analysis of p19^ARF^ in the inflammation foci showed a similar trend to that described above for p21^CIP1^, but with less accumulation of p19^ARF^‐positive cells in the lungs of D/Q‐treated mice (Figure 5c) (not statistically significant when analyzing individual mice and comparing groups; *p* = 0.0768, but significant when comparing total cells of each experimental group; *p* < 0.0001) (Figure 5d, Figure S6). Due to background problems with the anti‐p19^ARF^ antibody, quantification could not be performed in other areas of the lung.

When we analyzed another senescent marker such as p16^INK4a^, we observed a similar trend to that described above for p19^ARF^, although with lower percentages of positive cells (Figure S7a,b). Again, the percentage differences of total p16^INK4a^‐positive cells between the experimental groups, is significant (Figure S7c).

When we analyzed the percentage differences of positive cells for p21^CIP1^, p19^ARF^ and p16^INK4a^, of the SARS‐CoV‐2 infected groups, with respect to the mock group (Table S1), we observe the following: (i) on the one hand, the SARS‐CoV‐2‐dependent accumulation of these 3 markers follows a similar time‐course (always higher at 11 dpi), and (ii) on the other hand, and more importantly, that the mice belonging to D/Q + SARS‐CoV‐2 + D/Q group always presents less accumulation of these markers than the D/Q‐untreated infected group (except in the levels of p21^CIP1^ in parenchyma) (Table S1, see grey cells).

Thus, a preventive/therapeutic D/Q treatment clearly reduced the SARS‐CoV‐2‐dependent accumulation of the senescence markers p21^CIP1^, p19^ARF^, and p16^INK4a^ in the lungs.

### 2.8 A therapeutic challenge with senolytics

**FIGURE 6:**
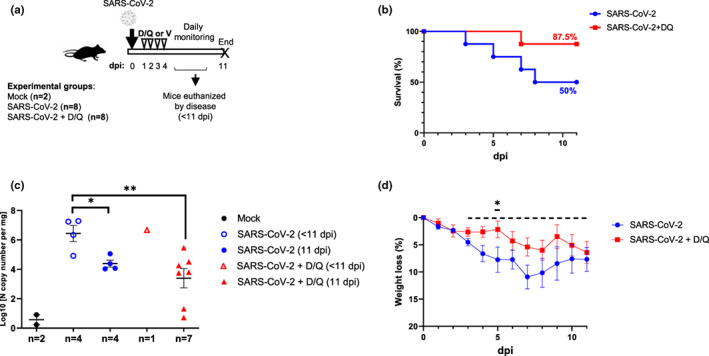
Scheme of the therapeutic challenge with senolytics and effect upon SARS‐CoV‐2 infection in K18‐hACE2 transgenic mice. (a) Experimental design for the therapeutic study. Mice (50% male/female) with a mean age of 12 months, were infected with SARS‐Cov‐2, and then treated with either D/Q or vehicle (V), and monitored as indicated in Methods. Dpi: Days post‐infection. (b) Percentage survival of mice. (c) Viral RNA levels in lungs analyzed by RT–qPCR. *p* Values were determined by one‐way ANOVA, Fisher's LSD test (*p = 0.0462; **p = 0.0027). (d) Weight change (±SEM) relative to day 0 was monitored (*N* of each group as indicated in [a]). The dashed line indicates the start of SARS‐CoV‐2‐induced deaths, and thus changing the *N* for each group. *p* Values were determined by two‐way ANOVA (uncorrected Fisher's LSD; mixed‐effects analysis) test (*p = 0.0439).

To further explore if a standalone therapeutic challenge would have a similar protective effect as the preventive/therapeutic one, we next tested early administration with D/Q (Figure 6a). One day after infection, the mice were treated with either D/Q or vehicle (SARS‐CoV‐2 + D/Q and SARS‐CoV‐2 groups, respectively) for 4 consecutive days (1–4 dpi; Figure 6a). The survival data showed an 87.5% (7/8) in the SARS‐CoV‐2 + D/Q group, in contrast to the 50% (4/8) in the SARS‐CoV‐2 group (Figure 6b). Although the protective effect of D/Q is evident, it was not significant (Gehan‐Breslow‐Wilcoxon test: *p* = 0.1092), unlike the preventive/therapeutic treatment shown in Figure 1 (*p* = 0.0398).

However, weight loss plots were very similar between the two experimental approaches (compare Figures 1d and 6d), reaching statistical significance in both cases at 5 dpi. In addition, viral load analysis in the lungs were also very similar between the two approaches (compare Figures 1c and 6c).

Lastly, we showed that SA‐β‐Gal (Senescence‐Associated β‐Galactosidase) activity is robustly accumulated in inflammation foci of lung tissue upon SARS‐CoV‐2 infection (Figure S8a), and importantly, it is significantly reduced in those surviving mice therapeutically treated with D/Q (Figure S8b). Thus, SA‐β‐Gal is another senescence marker that we detected upon SARS‐CoV‐2 infection, in line with the previous data with p16^INK4a^, p21^CIP1^, and p19^ARF^. The treatment with D/Q reduced the accumulation of these senescent markers (significantly in the case of p21^CIP1^ and SA‐β‐Gal), and hence the potential accumulation of senescent cells in lung tissue, which could be harmful at long term.

Therefore, a therapeutic administration of D/Q in response to a sub‐lethal dose of SARS‐CoV‐2, reduces mortality, shows significant two‐day delay in infection‐associated weight loss, significantly reduces the accumulation of the senescent marker SA‐β‐Gal, and increases the percentage of mice with reduced viral RNA burden.

## 3 DISCUSSION

Since the beginning of the COVID‐19 pandemic, there have been numerous studies warning of a possible pivotal role of senescent cells in the exacerbated immune response against SARS‐CoV‐2 (Blagosklonny, 2020; Kirkland & Tchkonia, 2020; Malavolta et al., 2020; Mohiuddin & Kasahara, 2021; Nehme et al., 2020). In this sense, a recent study (published during the course of our assays) using a related β‐CoV strongly suggested that the elimination of senescent cells by senolytics could be an alternative therapy for COVID‐19 disease (Camell et al., 2021).

Here, we have demonstrated that the senolytic drugs D/Q, are indeed efficient in improving response to SARS‐CoV‐2 infection, using susceptible K18‐hACE2 transgenic mice. The difference in statistical significance between the therapeutic assay and the preventive/therapeutic one may either highlight a protective effect of the preventive administration, or simply reflect the different N used in the assays. However, weight loss plots were very similar between the two experimental approaches (compare Figures 1d and 6d), reaching statistical significance in both cases.

We have shown that treatment with D/Q significantly increased SARS‐CoV‐2 survival, though differences in viral load and cytokine/chemokine levels were broadly associated with time of survival instead of specific to D/Q treatment. These data clearly suggest that the use of senolytics in the K18‐hACE2 mice increased survival/health‐associated parameters.

Viral load in the lungs was significantly lower in those mice who survived until 11 dpi (end of experiment), where the D/Q‐treated group was more represented. In our study of cytokines/chemokines related to severe COVID‐19 disease, survival was linked to lower levels as determined either by lung mRNA expression (*Il10*, *Oncostatin‐m*, *Il1α*, *Il1β*, *Cxcl10* and *Ccl2, Il4, Il4ra, Mrc1* and *Chi3l3*), or protein detection in serum (IFN‐γ, TNF‐α, IL‐1α, CCL‐2, CCL‐5). We hypothesize that senolytics increase the percentage of mice with reduced SARS‐CoV‐2‐derived symptoms through the Th2 cytokine *Il4* and its receptor *Il4ra*. This resulted in lower anti‐inflammatory M2 macrophage markers, which are associated with fibrosis and DAD (Boumaza et al., 2021; Vaz de Paula et al., 2020). In addition, the combination of senolytics D/Q enhanced *Ccl5* expression in the lungs, a feature reported to be associated with mild disease (Li et al., 2020; Zhao et al., 2020). In this sense, it is important to point out that the two animals from the D/Q + SARS‐CoV‐2 + D/Q (11 dpi) group showing the lowest *Ccl5* expression (Figure 3c), are the ones with the highest inflammation foci area and lung damage (Figure 4b). Moreover, previous reports in mice genetically deficient for CCL5 receptors, showed a most severe disease upon SARS‐CoV infection (Majumdar & Murphy, 2021), thus supporting a protective role of CCL5, as we observed here. Overall, our data can provide a link between monocyte/macrophage recruitment by CCL5 (Lee et al., 2017; Schall et al., 1990) and Th2 cytokine reduction (*Il4, Il4ra* and *Il10*), resulting in inhibition of M2 macrophage differentiation (*Mrc1* and *Chi3l3*), reduction of DAD and related morbidity. Our histopathological and symptoms analyses support this hypothesis, with less lung damage and dyspnea, respectively, observed in most of the surviving mice from the D/Q‐treated group.

Reduction in these disease markers was associated with a significant both delay in weight loss as well as a decreased burden of other clinical symptoms associated with COVID‐19, demonstrating a significant improvement in the overall health of mice treated with D/Q, and in agreement with previous reports (Palmer et al., 2019; Schafer et al., 2017; Sierra‐Ramirez et al., 2020; Zhang et al., 2019). It has been described in a murine model of fibrotic pulmonary disease that D/Q treatment improved pulmonary function and induced weight gain (Schafer et al., 2017). However, in this work we did not detect any weight gain, but rather a delay in the virus‐induced weight loss, suggesting the virus had more of a systemic impact that went beyond pulmonary disease.

Importantly, in response to SARS‐CoV‐2, we detected a prominent accumulation in the lung of cells positive for the senescence markers p21^CIP1^, p19^ARF^, p16^INK4a^ and SA‐β‐Gal. As far as we know, this is the first time that the accumulation of these four markers has been described in adult K18‐hACE2 mice, in response to SARS‐CoV‐2, and it followed a time‐dependent kinetic, with higher accumulation at 11 dpi (see Figure 5, Figures S7 and S8, Table S1).

In response to D/Q, this accumulation was either significantly (for p21^CIP1^ and SA‐β‐Gal) or substantially (for p19^ARF^ and p16^INK4a^) reduced, demonstrating on one hand the in vivo efficacy of the senolytics used in our study, and on the other hand, the association of lower levels of these markers with improved rates of mortality and morbidity.

The differences observed between the percentages of positive cells for each senescent marker (see Table S1), could be due either to an activation of specific senescent programs or to a senescent cell cleaning process dependent on each marker, as has been suggested in human tissues (Laura Idda et al., 2020).

It is known that even percentages as low as 1%–5% of chronic senescent cells can induce tissue dysfunction (McHugh & Gil, 2018). In our study, the lungs of adult (10 months old) mock‐infected K18‐hACE2 mice showed 0.3–0.6% of p21^CIP1^, p19^ARF^ and p16^INK4a^‐positive cells. However, in response to SARS‐CoV‐2 infection, these percentages increased about 10‐fold (average of the entire lung section analyzed), and even up to 77‐fold if considering the inflammation foci for p21^CIP1^‐positive cells (see Table S1 for a summary). Moreover, very recent works (published during the preparation of this article) reinforces our observations (Evangelou et al., 2022; Lee et al., 2021; Tsuji et al., 2022).

Tsuji et al. (2022) reported paracrine senescence induced by SARS‐CoV‐2, a result in the same line of our work. Although their in vivo work was carried out in experimental contexts different from ours, there are similarities with our data, such as: the accumulation of the senescence markers *p16* and *p19*, as well as the SASP (Senescence‐Associated Secretory Phenotype) factors *Ifnγ* and *Cxcl10*, in response to the infection. However, Tsuji et al. did not show either mortality nor apparent weight loss, which is clearly different to all the symptoms we described here. These differences are probably due to the different animal models and the strain of virus used.

By contrast, recently Lee et al. (2021) used a similar approach as we did, working with K18‐hACE2 mice and genuine SARS‐CoV‐2. They showed 100% survival in the mice treated with senolytics, whereas the infected, untreated group showed 60% of survival. These results were very similar to ours, although our experiments (with higher overall N) lasted longer, 11 dpi versus 6 dpi, which we believe better reflects the incidence of the SARS‐CoV‐2. In fact, it has been described that 11–12 dpi represents the whole spectrum of infection by SARS‐CoV (same family as of SARS‐CoV‐2), while 6 dpi is at the beginning of the second wave of cytokine/chemokine production (Chen et al., 2010), what supports our approach.

The immunohistological analysis of lung tissue by Lee et al. showed a SARS‐CoV‐2‐dependent accumulation of three senescent markers (p16^INK4a^, H3K9me3 and Lipofuscin), which are then reduced upon senolytic treatment, a result in the line of our data (with also p16^INK4a^, besides p19^ARF^, p21^CIP1^, and SA‐β‐gal).

In summary, the articles mentioned above (Camell et al., 2021; Evangelou et al., 2022; Lee et al., 2021; Tsuji et al., 2022), coincide with ours in the protective effect of senolytics, upon SARS‐CoV‐2 infection.

The SARS‐CoV‐2‐dependent increase in senescent cells (positive for p21^CIP1^ or p19^ARF^) between 7/8 dpi and 11 dpi that we detected, was significant (Figure 5b,d), and D/Q treatment directly reduced the percentage of these cells. However, it remains to be elucidated what percentage of the p21^CIP1^‐, p19^ARF^‐, p16^INK4a^ and SA‐β‐Gal‐positive cells in the lungs of the surviving infected mice would remain long‐term in the tissue, which may be the cause of post‐COVID‐19 syndrome‐associated pathologies that are currently being reported (Al‐Aly et al., 2021).

Based on the data shown, we hypothesize that SARS‐CoV‐2 increases the number of senescent cells in affected tissues, effectively accelerating the aging process, which may explain some of the post‐COVID‐19 sequelae described so far (Al‐Aly et al., 2021) (Figure S9). In fact, even in our limited experimental setting (11 dpi), we detected important pulmonary injuries in all surviving infected mice, suggesting medium/long‐term post‐COVID‐19 sequelae, such as pulmonary fibrosis. In this sense, more studies focusing on the long‐term effects of SARS‐CoV‐2 and their relation to senescence are necessary. We believe that a prolonged treatment with D/Q will ameliorate the long‐term lung damage observed in mice, although, it will be important to keep in mind other effects of D/Q administration apart from selectively killing senescent cells, when designing such assays (Ovadya & Krizhanovsky, 2018).

In summary, therapies focusing on the clearance of senescent cells, such as the one proposed here, hold exciting potential as emergent treatments for COVID‐19, both for short‐ and long‐term disease processes.

## 4 METHODS

### 4.1 Mice and ethics statement

The mice used in these experiments were obtained from the Jackson Laboratory (SN34860‐B6. Cg‐Tg(K18‐hACE2)2Prlmn/J). The original colony was expanded in our facility to produce the experimental cohort. Hemizygous animals were bred with C57BL6/J wt mice and offspring was genotyped according to Jackson's Separated PCR Assay. Animal experiment approval was provided by the Animal Protection Area of the Community of Madrid (CAM; PROEX reference number: 274.4/20).

### 4.2 Virus

SARS‐CoV‐2 virus (isolate NAVARRA‐2473) was obtained from the nasal swab of a COVID‐19 patient who had been hospitalized in the University of Navarra Clinic (Pamplona, Spain) in April 2020, with informed consent and after acquiring the necessary Regional Government permits. Initially, the virus sample was used to infect Vero‐E6 cells (ATCC® CRL‐1586™) which were grown with Eagle's minimum essential medium containing 10% fetal bovine serum and antibiotics. Cell supernatants were collected at 48 h post‐inoculation and titrated using a lysis plate assay in Vero‐E6 cell monolayers, resulting in a titer of 4.3 × 10^7^ plaque forming units (PFU)/ml. The viral stock was quickly frozen in a methanol‐dry ice bath and stored at −80°C. For subsequent experiments, smaller aliquots of the original stock were prepared, which after being frozen again had a titer of 1.8 × 10^7^ PFU/ml.

### 4.3 Senolytics

The senolytic drugs dasatinib (D) and quercetin (Q) were purchased from Selleckchem (Ref. S1021 and S2391, respectively), combined and diluted in propylene glycol (PG, Sigma, Ref. P4347), and sonicated in an Elmasonic P bath sonicator, at 37 kHz for 1 min, and then frozen at −20°C in aliquots corresponding to each day of therapy. On each day of treatment, the corresponding aliquot was thawed and saline solution (0.9% NaCl) was added to obtain the desired final volume. Subsequently, D/Q was administered by oral gavage at a concentration of 12 mg/kg (D) and 50 mg/kg (Q), in a volume of 100 μl/mouse. Two treatments were carried out with senolytics, preventive on days −20 to −16 (5 consecutive days) and therapeutic on days +1 to +4 (4 consecutive days) post‐infection. The control group was inoculated with PG by oral gavage.

### 4.4 Animal experiments

Transgenic K18‐hACE2 mice (age and sex as indicated in figure legends) were inoculated intranasally with 25 μl modified Eagle medium (MEM) or PBS, containing the indicated amounts of SARS‐CoV‐2. Mouse weight and health were monitored daily, up to 11 dpi (end of experiment) or until they reached parameters of severe disease (end‐point criteria) such as weight loss (≥20%), dyspnea, lethargy, piloerection, hunched posture, eye closure and staggering.

All experiments with SARS‐CoV‐2 were performed in BSL3 laboratories of the following animal care facilities: Veterinary Health Surveillance Center (VISAVET; Complutense University of Madrid), and the Centro de Biología Molecular Severo Ochoa (CBMSO‐CSIC, UAM).

### 4.5 Lung extraction

The right lung was inflated with formalin (4%) (Merck; Ref. 1004960700) then fixed with the same buffer for 7 days before being embedded in paraffin for histological tests. Lung tissue processing for SA‐β‐Gal staining of the therapeutic assay, was as follows: Half right lung was washed with PBS 1×, and then fixed with glutaraldehyde (0.2%) and paraformaldehyde (2%) in PBS 1×, for 1 hour at room temperature. Then the samples were washed with PBS 1× and frozen at −80°C until its processing. Then, the SA‐β‐Gal activity was performed as previously described (Sierra‐Ramirez et al., 2020). The left lung lobe was used for RNA extraction (cellular and viral), as described below.

### 4.6 RNA extraction and qRT‐PCR

For RNA extraction, lung tissue samples were incubated with either RNAlater Stabilization Solution (Thermo Fisher; Ref. AM7021) or TRI Reagent (Sigma Aldrich; Ref. T9424), and subsequently either frozen at ‐80 °C, or processed directly according to the RNeasy Plus Micro kit (Qiagen; Ref. 74034) or TRI Reagent ® RNA isolation reagent protocols, respectively. For extraction of viral RNA by nasal swabs, samples were collected at 4 dpi and processed using a MagMax core kit (Applied Biosystems; Ref. A32702) according to the manufacturer's instructions. Quantitative real‐time PCR (qPCR) was performed on purified RNA (lung or swab) to specifically detect SARs‐CoV‐2 RNA, according to the indications of the COVID‐19 dtec‐RT‐qPCR test kit (Genetic PCR Solutions; F100 format) (Figure 1c), or CBMSO Genomics and NGS Core Facility (GENGS), based on the “GENGS‐3V2F SARS‐CoV‐2 RT‐qPCR assay” (protected by CSIC as trade secret [5723–2020]) (Figure 6c), respectively.

For detection of cytokine and chemokine mRNA, samples were reverse transcribed using random priming and High‐capacity cDNA RT (Applied Biosystem), according to the manufacturer's instructions. qPCR was performed using DNA master SYBR Green I mix (Promega) in a QuantStudio thermocycler, and product quantities were calculated by applying the ΔC_t_ method ΔC_t_ = (C_t_ of gene of interest − C_t_ of housekeeping genes). The housekeeping genes used for input normalization were *β‐actin* and *36b4* for mice, and the following primer sequences were used:

**Table:** 

Mm*‐β‐Actin*	Fwd: GGACCACACCTTCTACAATG
	Rvs: GTGGTGGTGAAGCTGTAGCC
Mm‐*36b4*	Fwd: AGATTCGGGATATGCTGTTGG
	Rvs: AAAGCCTGGAAGAAGGAGGTC
Mm‐*Il10*	Fwd: AAGGACCAGCTGGACAACAT
	Rvs: TCATTTCCGATAAGGCTTGG
Mm‐Oncostatin	Fwd: GAGTACCAGGACCCAGTATGC
	Rvs: TGCTCAGGATGAGGAGACTGA
Mm‐*Il1α*	Fwd: AAGTCTCCAGGGCAGAGAGG
	Rvs: CTGATTCAGAGAGAGATGGTCAA
Mm‐*Il1β*	Fwd: AAAAGCCTCGTGCTGTCG
	Rvs: AGGCCACAGGTATTTTGTCG
Mm‐*Cxcl10*	Fwd: GGATCCCTCTCGCAAGGAC
	Rvs: CGTGGCAATGATCTCAACAC
Mm‐*Ccl2*	Fwd: CCACTCACCTGCTGCTACTC
	Rvs: GGACCCATTCCTTCTTGGGG
Mm‐*Tnfα*	Fwd: GCCTCTTCTCATTCCTGCTT
	Rvs: CTCCTCCACTTGGTGGTTTG
Mm‐*Ifnγ*	Fwd: CCTCATGGCTGTTTCTGGCT
	Rvs: CCTTTTGCCAGTTCCTCCAGA
Mm‐*Ccl5*	Fwd: CATATGGCTCGGACACCAC
	Rvs: CCTTCGAGTGACAAACACGA
Mm‐*Il4*	Fwd: CCAAACGTCCTCACAGCAAC
	Rvs: TTGGAAGCCCTACAGACGAG
Mm‐*Il13*	Fwd: AACGGCAGCATGGTATGGAGTG
	Rvs: TGGGTCCTGTAGATGGCATTGC
Mm‐*Il4ra*	Fwd: CCTGGAGTGAGTGGAGTCCTA
	Rvs: CAGGCAAAACAACGGGATGC
Mm‐*Mrc1*	Fwd: TTCAGAGGGGTTCACCTGGA
	Rvs: TTCAGAGGGGTTCACCTGGA
Mm‐*Chi3l3*	Fwd: TACTCACTTCCACAGGAGCAGG
	Rvs: CTCCAGTGTAGCCATCCTTAGG
Mm‐*Retnla*	Fwd: GCTGATGGTCCCAGTGAATACT
	Rvs: CACAAGCACACCCAGTAGCA
Mm‐*Arg1*	Fwd: AGCACTGAGGAAAGCTGGTC
	Rvs: CAGACCGTGGGTTCTTCACA
Mm‐*Cdkn1a‐p21*	Fwd: TCCACAGCGATATCCAGACA
	Rvs: GGACATCACCAGGATTGGAC

### 4.7 Histological processing

The samples fixed in 10% neutral buffered formalin solution (Panreac Química, SLU) were mounted in synthetic paraffin with a melting point of 56 °C (Casa Álvarez Material Científico), using a Citadel 2000 Tissue Processor (Thermo Fisher Scientific), with an automatic program applying alcohols of increasing concentration and xylene substitute (Citrus Clearing Solvent, Thermo Fisher Scientific). Blocks were made in a cold plate block forming unit (Histo Star Embedding Workstation, Thermo Fisher Scientific). Histological sections were obtained with a rotary microtome (Finesse Me+ Microtome, Thermo Fisher Scientific) at 3–4 μm thickness. A Gemini AS Automated Slide Stainer (Thermo Fisher Scientific) was used to stain the sections with hematoxylin–eosin and finally mounted using a CTM6 Coverslipper (Thermo Fisher Scientific), with a xylene‐based mounting medium (ClearVue Mountant, Thermo Fisher Scientific).

### 4.8 Immunohistochemistry (IHC)

Tissue samples were cut at 3 μm thickness, mounted on superfrost®plus slides and dried overnight. For IHC, an automated immunostaining platform was used (Autostainer Link, Dako or Ventana Discovery ULTRA; Roche). Antigen retrieval was performed with CC1 32 min, only for p21 and High pH buffer, Dako, Agilent (p19 and SARS‐CoV‐2 nucleocapsid); endogenous peroxidase was blocked (hydrogen peroxide at 3%) and slides were then incubated with the appropriate primary antibody as detailed: rat monoclonal anti‐p21^CIP1^ (291H; 1/10; CNIO, Monoclonal Core Unit); rat monoclonal anti‐p19^ARF^ (5‐C3‐1; 1/50; Santa Cruz sc‐32748); rat monoclonal anti‐CDKN2A/p16^INK4a^ antibody (33B; CNIO, Monoclonal Core Unit) and rabbit monoclonal anti‐SARS‐CoV‐2 nucleocapsid (N) protein (019; 1/15,000; Sino Biological 40143‐R019). After the primary antibody, slides were incubated with the corresponding secondary antibodies as needed (rabbit anti‐rat vector; visualization systems Rat on mouse HRP polymer Biocare Medical and Envision Rbb + Avidin Biotin). Immunohistochemical reaction was developed using 3,30‐diaminobenzidine tetrahydrochloride (DAB) and nuclei were counterstained with Carazzi's hematoxylin. Finally, the slides were dehydrated, cleared and mounted with a permanent mounting medium for microscopic evaluation. Positive control sections known to be primary antibody‐positive were included for each staining run. Whole slides were acquired with a slide scanner (AxioScan Z1, Zeiss), and images captured with the Zen Blue Software (Zeiss).

### 4.9 Serum cytokine and chemokine levels

Serum was collected by submandibular bleeding in EDTA‐coated tubes, from the sacrificed animals, and levels of IL‐10, IL‐1α, IL‐6, CCL5 (Rantes), CCL2 (MCP‐1), IFN‐γ, TNF‐α, and GM‐CSF, were analyzed in a pre‐configured multiplex panel (Procartaplex 8 plex; eBioscience; Ref. PPX‐08; Bioplex 200, Biorad). The data were analyzed with the Analyst 1.0 program (eBioscience).

### 4.10 Macro for quantification of p21^CIP1^ , p19^ARF^ , p16^INK4a^ and SA‐β‐Gal activity in lung sections

In order to perform the image treatment and counting of p21^CIP1^, p19^ARF^ and p16^INK4a^‐positive cells as well as SA‐β‐Gal activity, from lung sections, we developed specific scripts for ImageJ/FIJI software with the following steps. First, the images were converted to RGB format, an essential step for carrying out the image treatment. Next, a region of the RGB image was manually selected, to which was applied a “Color Deconvolution” to obtain the different stains of the sample. The following process was carried out for each of the obtained stains: First, “Subtract Background” was applied to reduce the possible background of the sample, after which a signal selection was made by applying a threshold based on the Renyi Entropy algorithm. On the obtained mask, possible outliers were eliminated and particles that were still attached were separated by the Watershed segmentation method. Finally, the selection and counting of the different particles was carried out.

### 4.11 Statistical analysis

Statistical significance was determined using Prism 9.1.0 software for Windows (GraphPad Software). Statistical outliers were identified using the ROUT method with Q = 1% (GraphPad). Gehan‐Breslow‐Wilcoxon test, one‐way ANOVA (Fisher's LSD) test, two‐way ANOVA (uncorrected Fisher's LSD; mixed‐effects analysis) test, Fisher's exact test and Chi‐square test of independence, were performed as indicated at each figure legend.

## AUTHOR CONTRIBUTIONS

Design the project (R.V., M.F., N.G., M.B., C.S., P.J.F.M., and C.V.K.); Performance of experiments (A.P., A.R.B., A.S.R., J.M.S., J.M., A.I.d.Á., C.O., and M.A.R.); Data analysis (A.P., A.R.B., A.S.R., G.G.A., M.B., C.S., P.J.F.M., and C.V.K.); Wrote the manuscript (C.V.K.).

## FUNDING INFORMATION

This work was supported by grants from the Instituto de Salud Carlos III (ISCIII), COV20‐00755 (C.V.K.) and COV20‐00792 (M.B., M.A.R.), Apadrina la Ciencia (C.S., C.O., G.G.A.), the AECC Scientific Foundation, PRDMA18011PAST, SIRTBIO‐ LABAE18008FERN (P.J.F.M., A.P., A.S.R.), the RETOS projects Programme of Ministerio de Ciencia e Innovación (MICINN), SAF2017‐85766‐R and PID2020‐114077RB‐I00 (P.J.F.M., A.P., A.S.R.), Ramon y Cajal Award from MICINN, RYC‐2017‐22335 (P.J.F.M., A.P., A.S.R.), Proyecto REACT from REACT/European Union/FEDER, COVTRAVI‐19‐CM U (J.M.S., M.F., N.G.), grant from Consejo Superior de Investigaciones Científicas (CSIC), CSIC‐COV19‐014 (A.I.d.Á), PID2020‐113888RB‐I00 from MICINN (A.I.d.Á), and the European Commission–Next Generation EU (regulation EU 2020/2094), through the CSIC's Global Health Platform (PTI+ Global Health) (A.I.d.Á). The RT–qPCR reactions were provided by the Genomics and NGS Core Facility (GENGS) at the Centro de Biología Molecular Severo Ochoa (CBMSO, CSIC‐UAM) Madrid, Spain, which obtains general funding from both institutions. The GENGS facility is part of the PTI+ Global Health (CSIC) (http://www.cbm.uam.es/genomica).

## CONFLICT OF INTEREST

C.V.K. declares that he is co‐founder of SenCell Therapeutics S.L.

## Supporting information


Figures S1–S9

Appendix S1

## Data Availability

The data that support the findings of this study are available from the corresponding author upon reasonable request.
