# Supplementary material for: Treatment with the senolytics dasatinib/quercetin reduces SARS‐CoV‐2‐related mortality in mice
Source: Aging Cell. 2023 Jan 26;22(3):e13771. doi: 10.1111/acel.13771 (PMC10014049; doi:10.1111/acel.13771)
Supplement: Supplementary file 1 — Figures S1–S9 [file ACEL-22-e13771-s001.pdf]

Figure S1

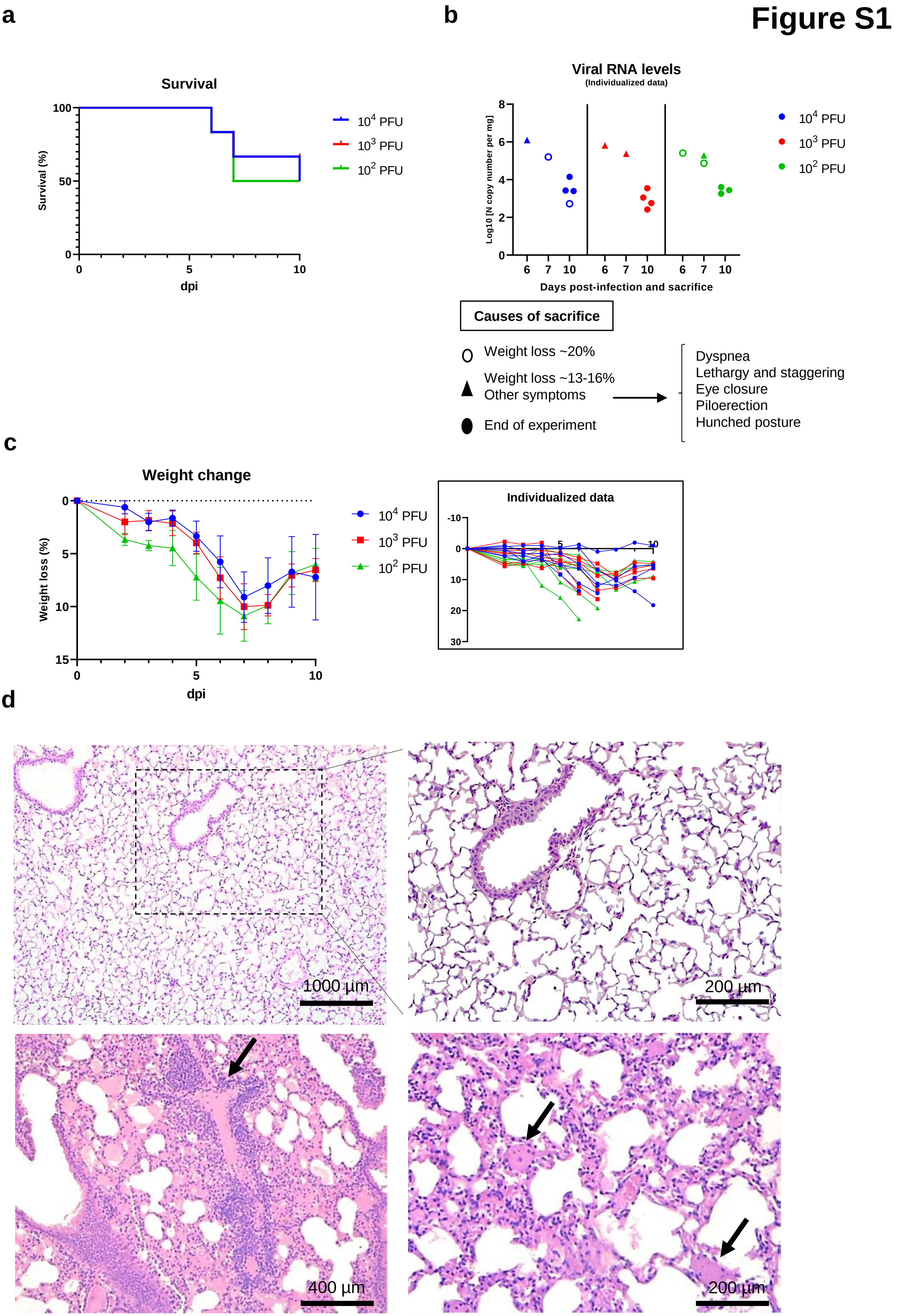
a
b
Survival
Survival (%)
100
50
0
0
5
10
dpi
10^4 PFU
10^3 PFU
10^2 PFU
Viral RNA levels
(Individualized data)
Log10 [N copy number per mg]
8
6
4
2
0
6
7
10
6
7
10
6
7
10
Days post-infection and sacrifice
10^4 PFU
10^3 PFU
10^2 PFU
Causes of sacrifice
Weight loss ~20%
Weight loss ~13-16%
Other symptoms
End of experiment
Dyspnea
Lethargy and staggering
Eye closure
Piloerection
Hunched posture
c
Weight change
Weight loss (%)
0
5
10
15
0
5
10
dpi
10^4 PFU
10^3 PFU
10^2 PFU
Individualized data
-10
0
10
20
30
5
10
d
1000 µm
200 µm
400 µm
200 µm

Figure S2

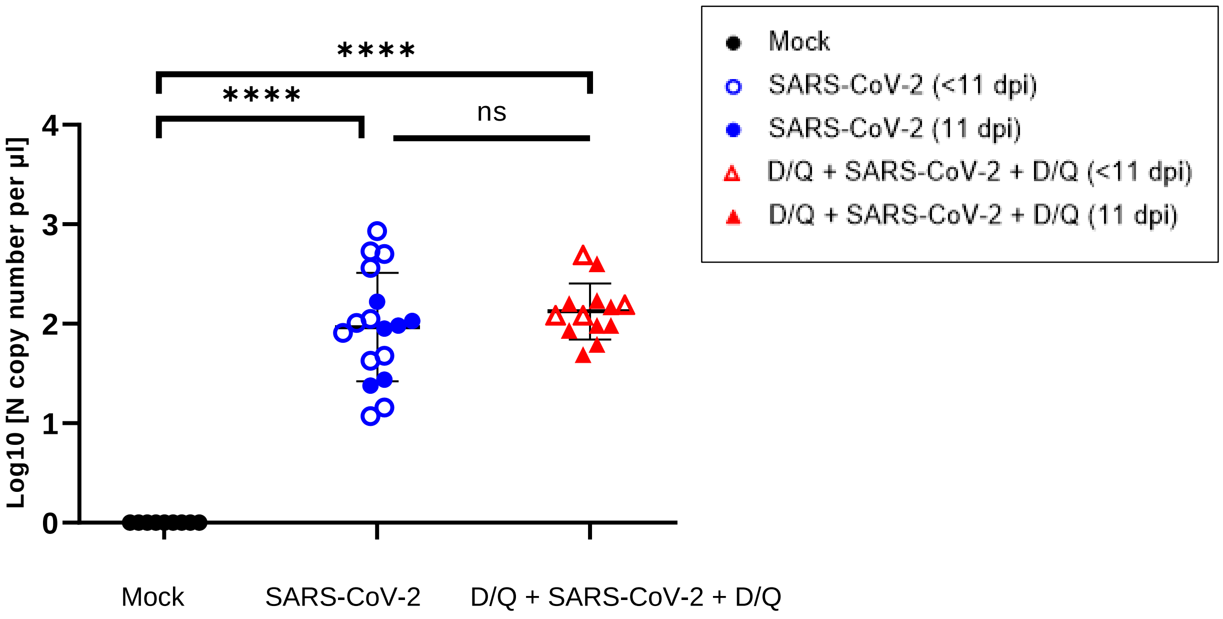

****
****
ns
Log10 [N copy number per µl]
4
3
2
1
0
Mock
SARS-CoV-2
D/Q + SARS-CoV-2 + D/Q
Mock
SARS-CoV-2 (<11 dpi)
SARS-CoV-2 (11 dpi)
D/Q + SARS-CoV-2 + D/Q (<11 dpi)
D/Q + SARS-CoV-2 + D/Q (11 dpi)

Figure S3

a

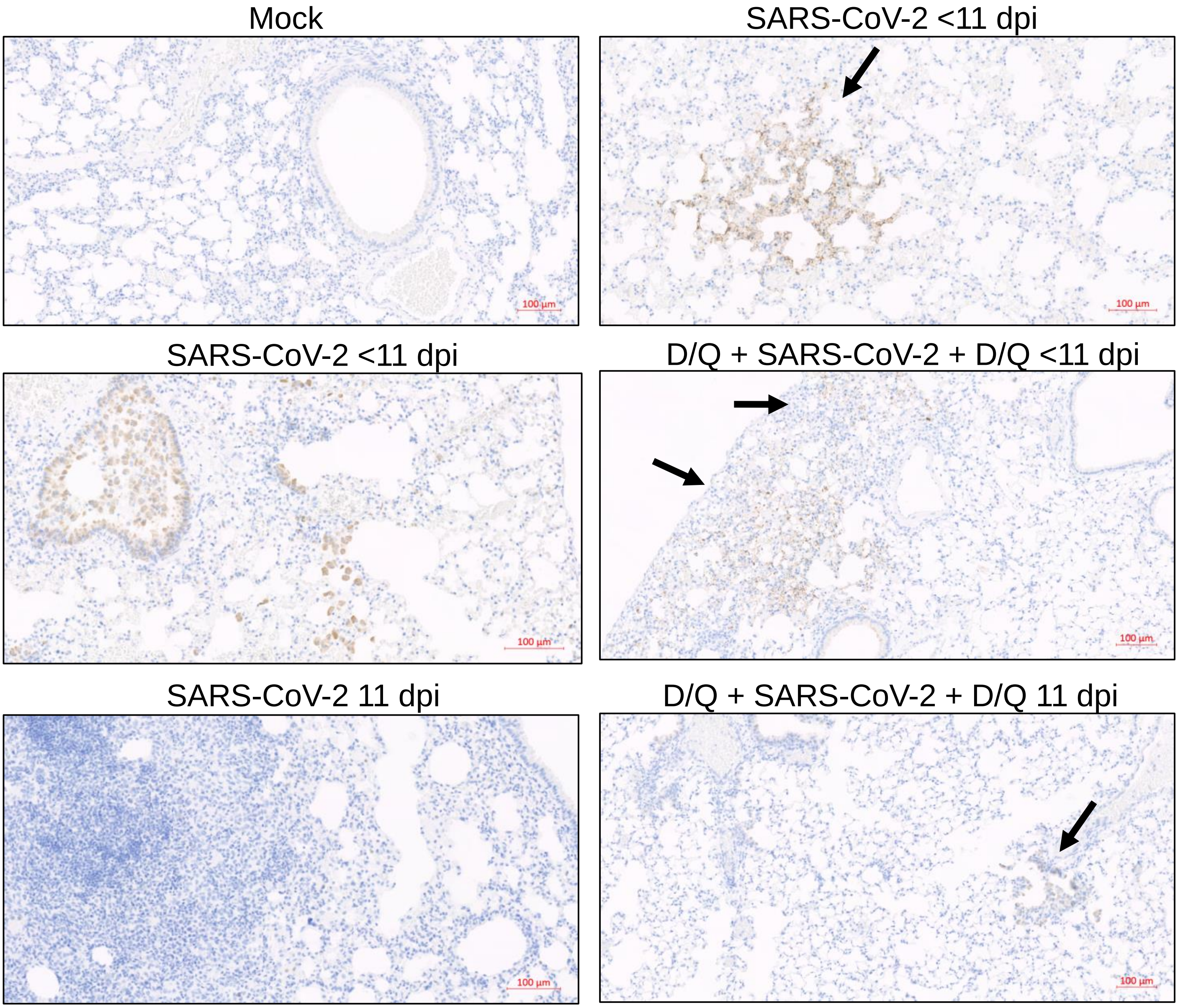
Mock
SARS-CoV-2 <11 dpi
SARS-CoV-2 <11 dpi
D/Q + SARS-CoV-2 + D/Q <11 dpi
SARS-CoV-2 11 dpi
D/Q + SARS-CoV-2 + D/Q 11 dpi
100 µm

b

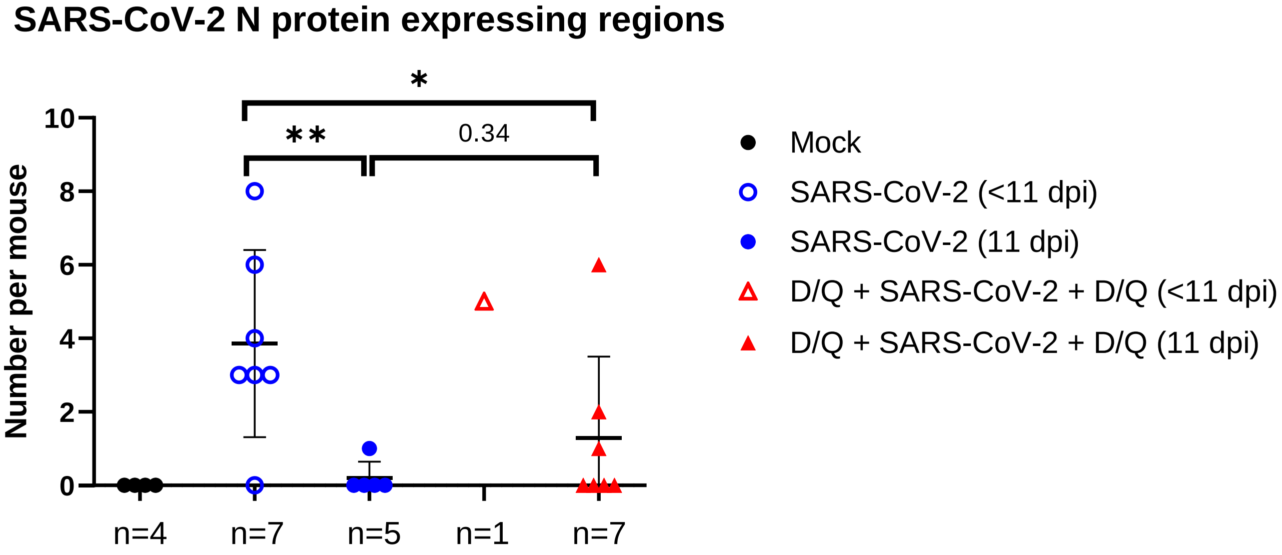
SARS-CoV-2 N protein expressing regions
Number per mouse
0
2
4
6
8
10
*
**
0.34
n=4
n=7
n=5
n=1
n=7
Mock
SARS-CoV-2 (<11 dpi)
SARS-CoV-2 (11 dpi)
D/Q + SARS-CoV-2 + D/Q (<11 dpi)
D/Q + SARS-CoV-2 + D/Q (11 dpi)

Figure S4

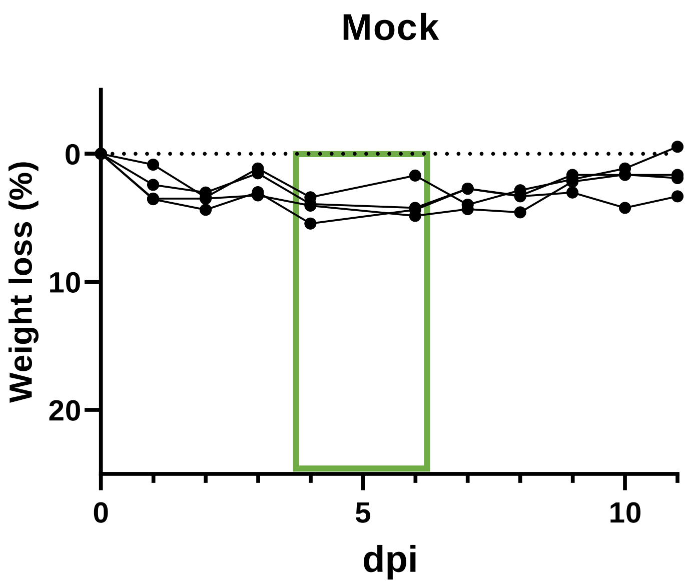
Mock
Weight loss (%)
0
10
20
0
5
10
dpi

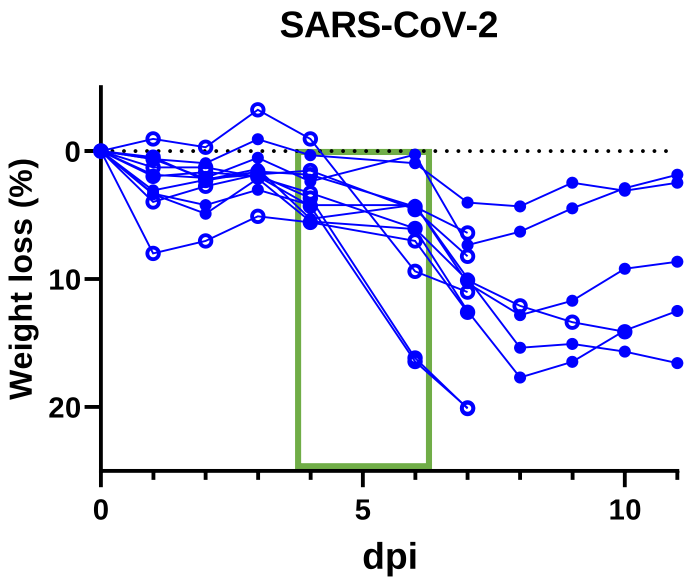
SARS-CoV-2
Weight loss (%)
0
10
20
0
5
10
dpi

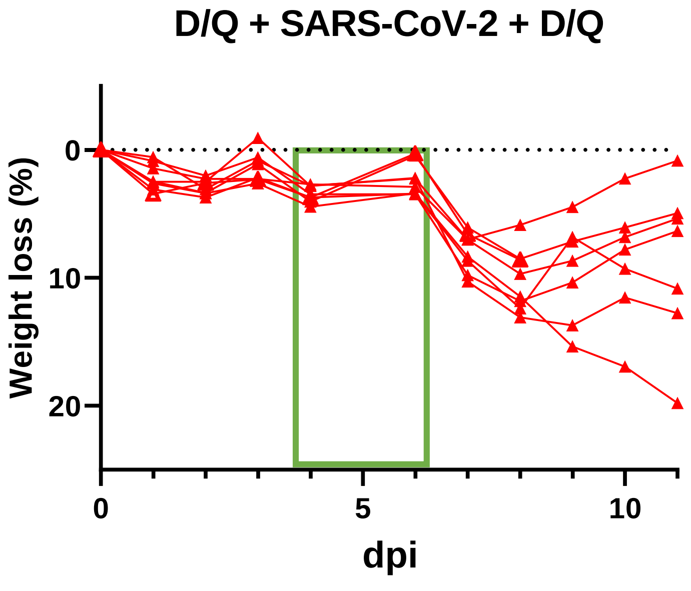
D/Q + SARS-CoV-2 + D/Q
Weight loss (%)
0
10
20
0
5
10
dpi

Figure S5

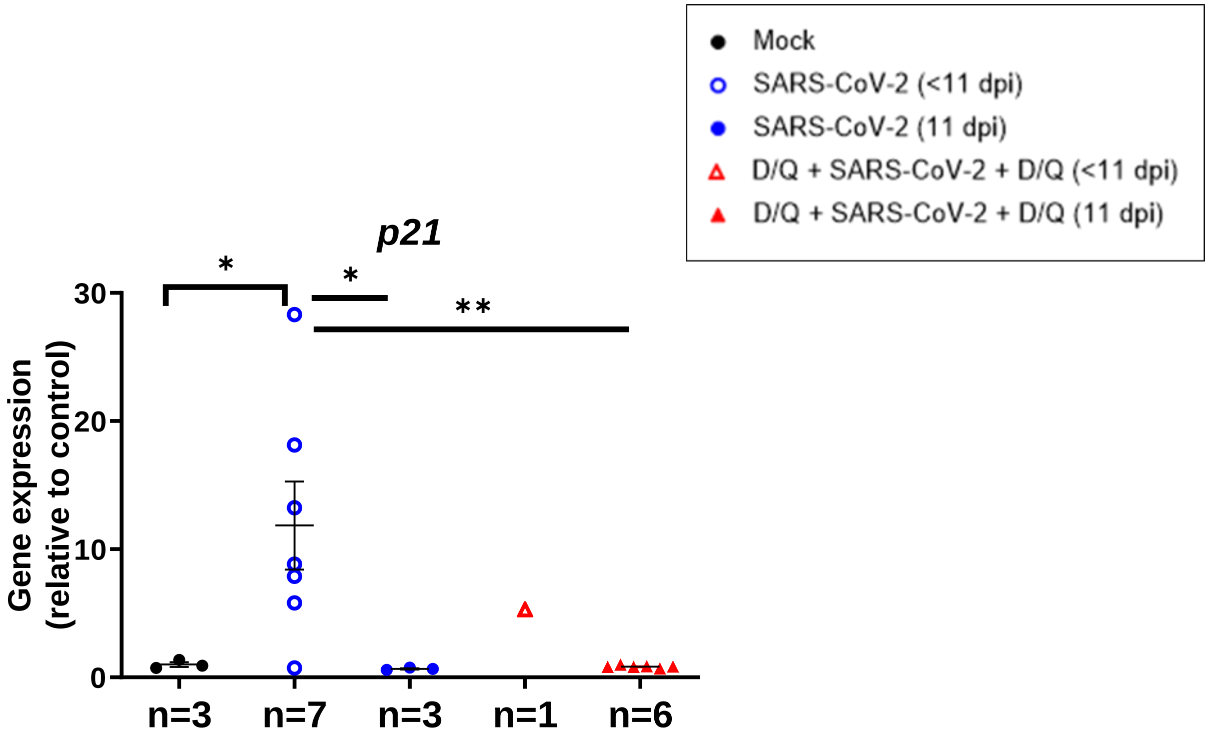

Mock
SARS-CoV-2 (<11 dpi)
SARS-CoV-2 (11 dpi)
D/Q + SARS-CoV-2 + D/Q (<11 dpi)
D/Q + SARS-CoV-2 + D/Q (11 dpi)
p21
Gene expression (relative to control)
30
20
10
0
*
*
**
n=3
n=7
n=3
n=1
n=6

Figure S6

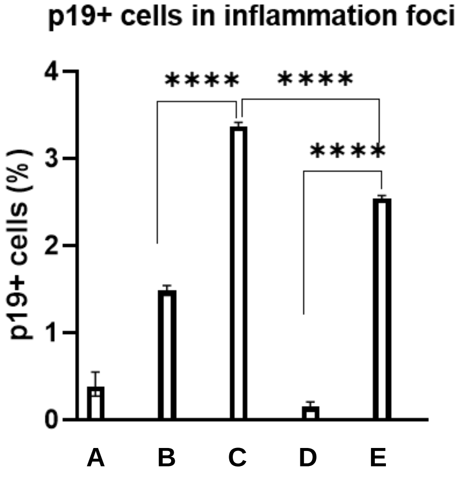

	p19+ cells	Total cells	p19+(%)
**A-** Mock	31	7.955	0.38
**B**- SARS-CoV-2 (<11 dpi)	2.656	178.621	1.48
**C**- SARS-CoV-2 (11 dpi)	22.091	654.979	**3.37**
**D**- D/Q+SARS-CoV-2+D/Q (<11 dpi)	35	23.179	0.15
**E**- D/Q+SARS-CoV-2+D/Q (11 dpi)	20.645	811.637	**2.54**

Figure S7

a

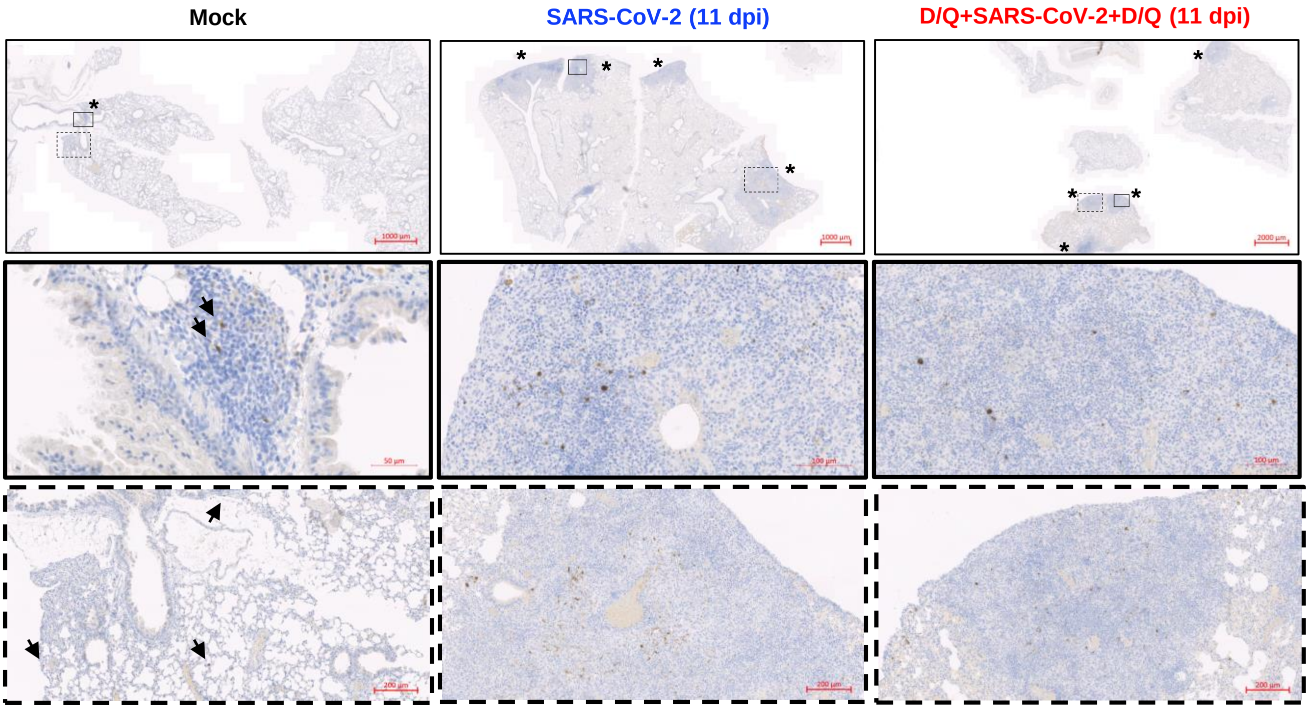

b

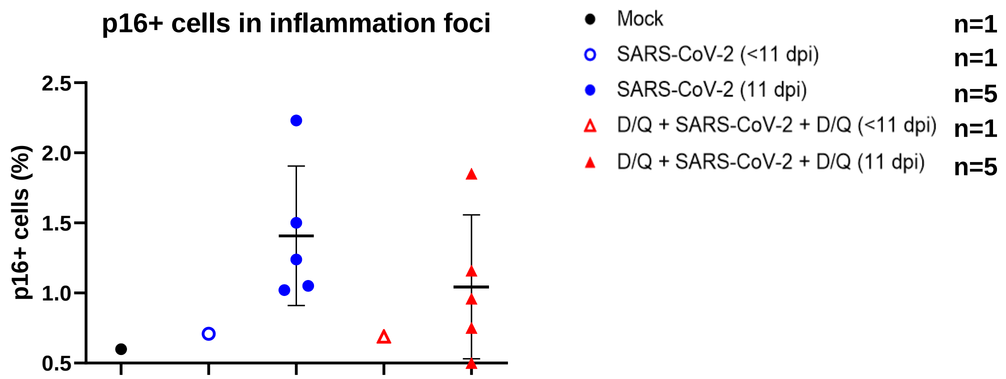

c

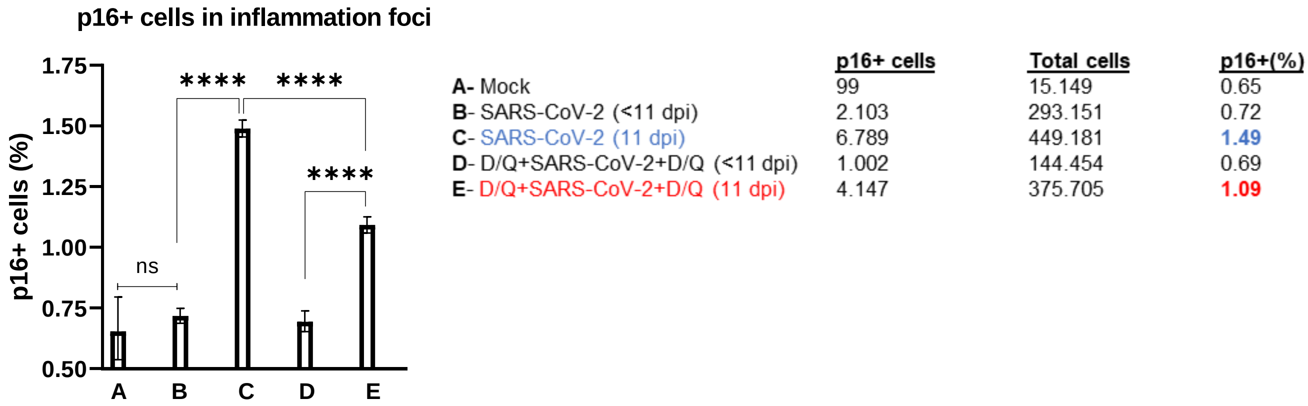

	p16+ cells	Total cells	p16+(%)
**A**- Mock	99	15.149	0.65
**B**- SARS-CoV-2 (<11 dpi)	2.103	293.151	0.72
**C**- SARS-CoV-2 (11 dpi)	6.789	449.181	**1.49**
**D**- D/Q+SARS-CoV-2+D/Q (<11 dpi)	1.002	144.454	0.69
**E**- D/Q+SARS-CoV-2+D/Q (11 dpi)	4.147	375.705	**1.09**

Figure S8

a

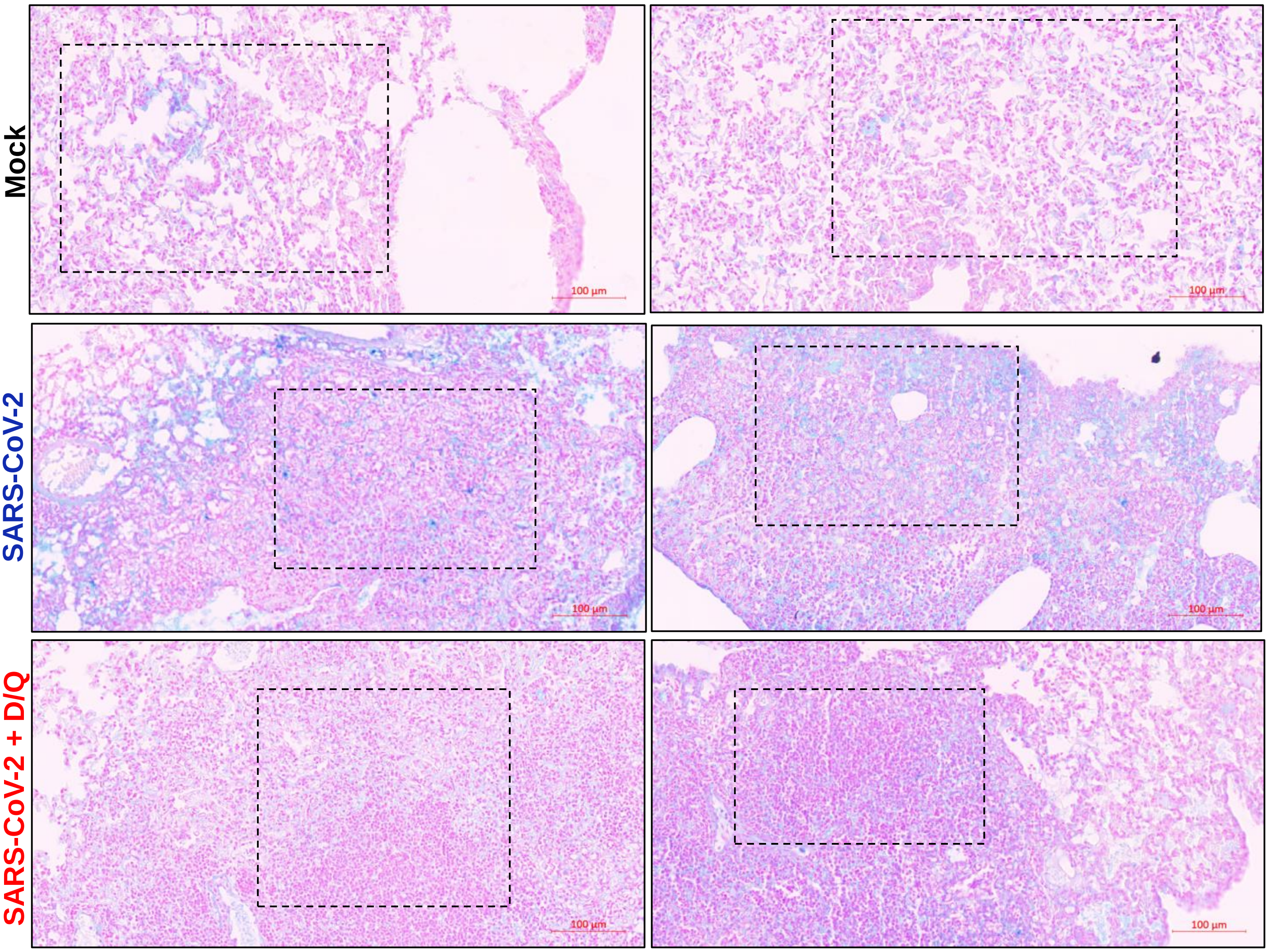

Mock
SARS-CoV-2
SARS-CoV-2 + D/Q
100 µm

b

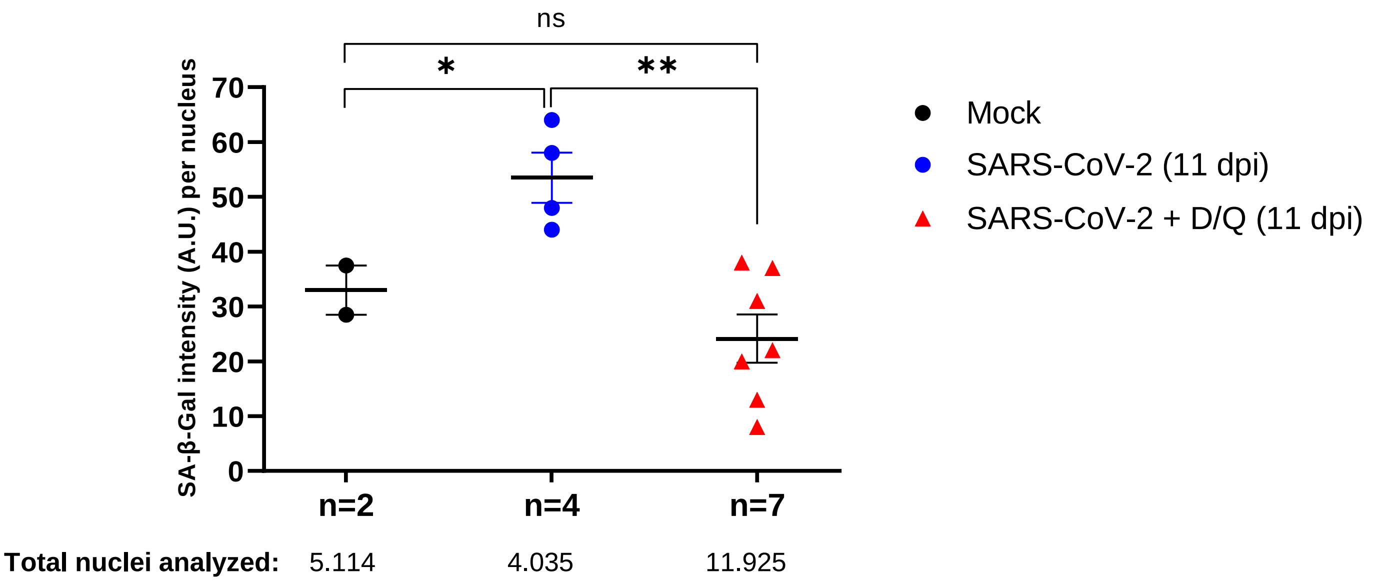

ns
*
**
SA-β-Gal intensity (A.U.) per nucleus
70
60
50
40
30
20
10
0
n=2
n=4
n=7
Mock
SARS-CoV-2 (11 dpi)
SARS-CoV-2 + D/Q (11 dpi)
Total nuclei analyzed:
5.114
4.035
11.925

Figure S9

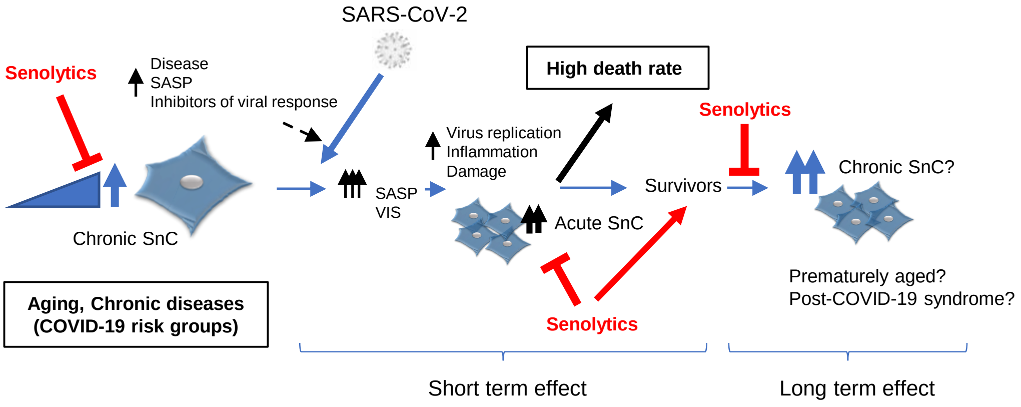
SARS-CoV-2
Senolytics
Disease
SASP
Inhibitors of viral response
High death rate
Senolytics
Virus replication
Inflammation
Damage
SASP
VIS
Survivors
Chronic SnC?
Chronic SnC
Acute SnC
Prematurely aged?
Post-COVID-19 syndrome?
Aging, Chronic diseases
(COVID-19 risk groups)
Senolytics
Short term effect
Long term effect
