# Supplementary material for: Treatment with the senolytics dasatinib/quercetin reduces SARS‐CoV‐2‐related mortality in mice
Source: Aging Cell. 2023 Jan 26;22(3):e13771. doi: 10.1111/acel.13771 (PMC10014049; doi:10.1111/acel.13771)
Supplement: Supplementary file 2 — Appendix S1 [file ACEL-22-e13771-s002.docx]

**SUPPLEMENTARY INFORMATION**

**Title**: Treatment with the senolytics dasatinib/quercetin reduces SARS-CoV-2-related mortality in mice.

**Running title**: Senolytics reduces COVID-19 mortality

**Authors**: Andrés Pastor-Fernández^1, 9^, Antonio R. Bertos^2, 9^, Arantzazu Sierra-Ramírez^1^, Javier del Moral-Salmoral^3, 4^, Javier Merino^3, 4^, Ana I. de Ávila^4, 5^, Cristina Olagüe^6^, Ricardo Villares^7^, Gloria González-Aseguinolaza^6^, María Ángeles Rodríguez^8^, Manuel Fresno^3, 4^, Nuria Gironés^3, 4^, Matilde Bustos^8^, Cristian Smerdou^6^, Pablo Jose Fernandez-Marcos^1^, Cayetano von Kobbe^4, 10, 11^

**Affiliations**:

^1^Metabolic Syndrome Group-BIOPROMET, Madrid Institute for Advanced Studies-IMDEA Food, CEI UAM+CSIC, E28049 Madrid, Spain.

^2^Department of Internal Medicine and Surgical Animal, Faculty of Veterinary/ VISAVET Centre, Complutense University of Madrid, Madrid, Spain.

^3^Departamento de Biología Molecular, Universidad Autónoma de Madrid (UAM), E28049 Madrid, Spain.

^4^Centro de Biología Molecular Severo Ochoa (CSIC-UAM), Consejo Superior de Investigaciones Científicas (CSIC), Campus de Cantoblanco, E28049 Madrid, Spain.

^5^Centro de Investigación Biomédica en Red de Enfermedades Hepáticas y Digestivas (CIBERehd) del Instituto de Salud Carlos III, 28029, Madrid, Spain.

^6^Division of Gene Therapy and Regulation of Gene Expression, CIMA Universidad de Navarra, 31008 Pamplona, Spain.

^7^Centro Nacional de Biotecnología (CNB-CSIC), Consejo Superior de Investigaciones Científicas (CSIC), E28049 Madrid, Spain.

^8^Institute of Biomedicine of Seville (IBiS), Spanish National Research Council (CSIC), University of Seville, Virgen del Rocio University Hospital, Seville, Spain.

^9^ These authors contributed equally to this work

^10, 11^ Lead contact and correspondence: [cvonkobbe@cbm.csic.es](mailto:cvonkobbe@cbm.csic.es)

**SUPPLEMENTAL FIGURES LEGENDS**

**Figure S1**. **SARS-CoV-2 titration assay**. Ten-month-old K18-hACE2 mice (males) were divided in three groups (n=6), and infected with the indicated amounts of SARS-CoV-2 per mouse. **a**. Percentage of survival of mice. **b**. Viral RNA levels analyzed by RT-qPCR (top). Cause of euthanasia and/or sacrifice (bottom). **c**. Weight change was monitored. Small square corresponds to individualized data. **d**. Hematoxylin and eosin staining of lung sections from non-infected (upper images) and SARS-CoV-2-infected (10^4^ PFU) (lower images) K18-hACE2 mice. Veterinary pathologists performed a blinded histological evaluation of the lung samples, scoring the lesions and diagnosing pneumonia as bronchiolo-interstitial, lymphocytic, multifocal, sub-acute to chronic, moderate to intense. However, the diffuse alveolar damage (DAD) and disseminated intravascular coagulation (DIC) were most intense in the group of mice receiving the highest dose (10^4^ PFU/mouse). Since intense DAD and DIC has been described in severe COVID-19 patients [1, 2], we chose to use 10^4^ PFU/mouse for subsequent experiments. Arrows show the presence of thrombi and emboli which induce hemorrhage. Scale bars as indicated in each image.

**Figure S2**. **Viral RNA levels in nasal swabs**. Samples were taken at 4 dpi from each mouse, and the RNA was isolated as indicated in methods. Mice groups and date of sacrificed as indicated in the figure. *P* values were determined by one-way ANOVA, Fisher´s LSD test. ns: not significant; ****: *P* ≤ 0.0001. Data are combined from two independent experiments.

**Figure S3**. **Immunohistochemistry (IHC) staining images of SARS-CoV-2 nucleocapside protein (N) in lung tissues of representative mice from each group**. **a**. Representative lung tissue samples from the following groups: Mock, SARS-CoV-2 (euthanized <11 dpi, and sacrificed at 11 dpi), D/Q+SARS-CoV-2+D/Q (euthanized <11 dpi, and sacrificed at 11 dpi), as indicated in the figure. Arrows: representative foci with expression of SARS-CoV-2 N protein. Scale bars: 100 µm. **b**. Quantification of the number of SARS-CoV-2 N protein expressing foci per mouse of each experimental group. *P* values were determined by one-way ANOVA, Fisher´s LSD test. *: *P* ≤ 0.05; **: *P* ≤ 0.005. Data from one out of two independent experiments.

**Figure S4**. **Weight loss individualized data**. Weight change data for each mouse from the indicated experimental groups. Green rectangle: two-day window in which the mock and D/Q+SARS-CoV-2+D/Q groups showed identical responses, unlike the group infected by SARS-CoV-2 alone. Data are representative over two independent experiments.

**Figure S5**. **mRNA expression of p21 in lung**. *P* values were determined by one-way ANOVA, Fisher´s LSD test. ns: not significant. *: *P* ≤ .05; **: *P* ≤ .005. Data from one out of two independent experiments.

**Figure S6. Quantification of total p19^ARF^-positive cells of each analyzed experimental group**. Bottom table: numerical data of the number of cells analyzed from the indicated groups. *P* values determined by Fisher´s exact test (****: *P*≤ .0001). The Chi-square with Yates' correction was calculated with large samples. Data from one out of two independent experiments.

**Figure S7**. **Immunohistochemistry (IHC) staining images of p16^INK4a^ in lung tissues from SARS-CoV-2 infected K18-hACE2 mice. a.** IHC of p16^INK4a^-positive cells of representative lung samples from mice of the indicated groups. Top row: low magnification of representative lung tissues. Asterisks (*) indicate areas of lymphocyte accumulation (mock group) or dense inflammation foci (SARS-CoV-2 infected groups), where the quantification of the p16^INK4a^-positive cells has been carried out (as indicated in b). Squares with solid or broken lines, indicate the magnified regions shown in both the middle and the bottom rows, corresponding to representative areas. Arrows indicate p16^INK4a^-positive cells in mock sample. Scale bars: top row (from left to right): 1000 µm, 1000 µm and 2000 µm. Middle row (from left to right): 50 µm, 100 µm and 100 µm. Bottom row: 200 µm. **b**. Quantification of p16^INK4a^-positive cells of lung samples from individually analyzed mice, using ImageJ/FIJI software, from the regions marked by asterisks in a, as well as from lung regions in representative mice sacrificed <11 dpi (which do not show dense inflammation foci). **c**. Quantification of total p16^INK4a^-positive cells of each experimental group analyzed in b. Bottom table: numerical data of the number of cells analyzed from the indicated groups. *P* values determined by Fisher´s exact test (****: *P*≤ .0001). The Chi-square with Yates' correction was calculated with large samples. Data from one out of two independent experiments.

**Figure S8.** **SA-β-Gal (Senescence-Associated β-Galactosidase) activity in lung samples from SARS-CoV-2-surviving mice**. **a**. Representative lung tissue samples from the following groups: Mock (n=2), SARS-CoV-2 (sacrificed at 11 dpi; n=4), SARS-CoV-2+D/Q (sacrificed at 11 dpi; n=7), as indicated in the figure. Dotted square indicates selected area for quantification with the specific macro (avoiding the background signal in both the bronchial epithelium and areas without nuclei displaying X-Gal precipitation). Scale bars: 100 µm. **b**. Quantification of the SA-β-Gal activity (blue stain intensity) in selected areas of tissue, using an ad hoc ImageJ/FIJI software. The mean value of the intensity/activity (represented as A.U.) was divided by the number of nuclei, to represent the activity per nucleus/cell. Total number of nuclei analyzed from each experimental group are indicated. *P* values were determined by one-way ANOVA, Fisher´s LSD test. *: *P* = 0.0478; **: *P* = 0.0012.

**Figure S9**. **Senescent cells (SnC) are central targets for treatment of COVID-19**. Schematic of the working hypothesis generated from the data presented herein. COVID-19 risk groups display a high burden of chronic SnC, and the corresponding increase in secreted pro-inflammatory factors (SASP; senescence-associated secretory phenotype), which favor SARS-CoV-2 infection. In this scenario, mainly due to both a bystander effect and VIS (virus-induced senescence), there will be a significant increase of acute SnC, which will consequently exacerbate the inflammatory response. In survivors, over time these acute SnC will become chronic, which could explain some of the symptoms of post-COVID-19 syndrome. The senolytics dasatinib and quercetin could be promising treatments for COVID-19, both short- and long-term. Likewise, a preventive treatment would reduce the burden of SnC, reducing the impact of SARS-CoV-2 infection.

**Table S1. Summary of the fold differences in p21^CIP1^, p19^ARF^ and p16^INK4a^-**

**positive cells in lung tissue by IHC**.

|  | p21^CIP1^ ***** | | | p19^ARF^ | p16^INK4a^ |
| --- | --- | --- | --- | --- | --- |
| Experimental groups | Lung section | Parenchyma | Inflammation foci | | |
| Mock | 1 | 1 | 1 | 1 | 1 |
| SARS-CoV-2 (<11 dpi) | 3.6 | 2.59 | 39.18 | 3.89 | 1.1 |
| DQ+SARS-CoV-2+DQ (<11 dpi) | 3.45 | 3.03 | 19 | 0.39 | 1.06 |
| SARS-CoV-2 (11 dpi) | 12.6 | 6.4 | 77.36 | 8.86 | 2.29 |
| DQ+SARS-CoV-2+DQ (11 dpi) | 8.93 | 3.84 | 51.36 | 6.68 | 1.67 |

*Number of analyzed samples: 24 (for p21^CIP1^ and p19^ARF^) and 13 (for p16^INK4a^).

Gray cells: higher values when comparing SARS-CoV-2 and DQ+SARS-CoV-2+DQ groups, sacrificed before (< 11 dpi) or at the end-point (11 dpi).

dpi: days post-infection

**References**

1. Tang, N. et al. Abnormal coagulation parameters are associated with poor prognosis in patients with novel coronavirus pneumonia. *J Thromb Haemost.* **18**, 844–847. <https://doi:10.1111/jth.14768> (2020).
2. Menter, T. et al. Postmortem examination of COVID‐19 patients reveals diffuse alveolar damage with severe capillary congestion and variegated findings in lungs and other organs suggesting vascular dysfunction. *Histopathology*. **77**, 198–209. <https://doi:10.1111/his.14134> (2020).
